# A New In Situ Prepared MOF‐Natural Polymer Composite Electrolyte for Solid Lithium Metal Batteries with Superior High‐ Rate Capability and Long‐Term Cycling Stability at Ultrahigh Current Density

**DOI:** 10.1002/advs.202203916

**Published:** 2022-11-15

**Authors:** Jiazhu Guan, Xinping Feng, Qinghui zeng, Zhenfeng Li, Yu Liu, Anqi Chen, Honghao Wang, Wei Cui, Wei Liu, Liaoyun Zhang

**Affiliations:** ^1^ School of Chemical Sciences University of Chinese Academy of Sciences Beijing 100049 P. R. China

**Keywords:** composite electrolyte, high‐energy‐density solid lithium batteries, inhibiting lithium dendrite, metal‐organic frameworks, natural polymer

## Abstract

Lithium metal batteries hold promise for energy storage applications but suffer from uncontrolled lithium dendrites. In this study, a new composite membrane based on modified natural polymer and ZIF‐67 is designed and prepared by the in situ composite method for the first time. Among them, a modified natural polymer composed of lithium alginate (LA) and polyacrylamide (PAM) can be obtained by electrospinning. Importantly, the polar functional groups of natural polymers can interact by hydrogen bonding and MOFs can construct lithium‐ion transport channels. Consequently, compared with LA‐PAM electrolyte without MOF, the electrochemical stability window of ZIF‐67‐LA‐PAM electrolyte becomes wider from 4.5 to 5.2 V, and the lithium‐ion transference number (*t*_Li+_) enhances from 0.326 to 0.627 at 30°C. It is worth noting that the symmetric cells with ZIF‐67‐LA‐PAM have superior stable cycling performance at 40 and 100 mA cm^−2^, and a high rate at 10C and 20C for LFP cells. Besides, the cell with NCM811 high‐voltage cathode can run stably for 400 cycles with an initial discharge capacity of 136.1 mAh g^−1^ at 0.5C. This work provides an effective method for designing and preparing MOF‐natural polymer composite electrolytes and exhibits an excellent application prospect in high‐energy‐density lithium metal batteries.

## Graphical abstract

A natural polymer composite electrolyte is prepared by in situ synthesis of ZIF‐67 on a modified lithium alginate electrospinning membrane. The assembled cells can run stably for 1300 h at 100 mA cm^−2^ for symmetric cells, 1600 cycles at 20C for LFP cells, and even 400 cycles at 2C for pouch cells. The obtained electrolyte can also well adapt to the NCM811 electrode.

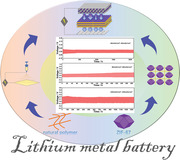

## 1 Introduction

Lithium metal is a promising anode material for lithium batteries due to its high specific capacity (3860 mAh g^−1^) and low redox potential (−3.04 V vs the standard hydrogen electrode).^[^ ^1^ ^]^ However, lithium dendrites caused by uneven lithium deposits during the lithium plating/stripping process would easily lead to a short circuit of cells and decrease the specific capacity of lithium batteries.^[^ ^2^ ^]^ Consequently, it has become a challenge to inhibit the formation and growth of lithium dendrites for solving the safety and improving the performance of lithium metal batteries.

Constructing ion transport channel is an effective method to suppress the lithium dendrites’ growth, which is conducive to reducing ion concentration polarization and speeding up the homogenization of lithium‐ion deposition.^[^ ^3^ ^]^ Metal‐organic‐framework materials (MOFs) assembled by metal ions or inorganic clusters and organic ligands^[^ ^4^ ^]^ are a kind of porous material with a large specific surface area. Owing to the unique structure, MOFs can absorb numerous liquid electrolytes and impurities, as well as can coordinate with anions by the unsaturated metal sites to promote Li^+^ transmission.^[^ ^5^ ^]^ More importantly, the periodical crystalline and ordered channels may provide a lithium‐ion transport pathway for homogeneous Li^+^ deposition.^[^ ^2^, ^6^ ^]^ Recently, the application of MOFs in electrolytes for lithium batteries have attracted much attention. Diandian Han et al.^[^ ^6^ ^]^ used ZIF‐8 to prepare a gel solid electrolyte, which showed a good ability to suppress lithium dendrites and a high Li^+^ transference number of 0.68. Wenyan Shang et al.^[^ ^5a^ ^]^ used UIO‐66‐NH_2_ as an additive to prepare a solid electrolyte, which exhibited a wider electrochemical window of 5.4 V and a better ability to suppress lithium growth. Xuewei Fu et al.^[^ ^7^ ^]^ combined UIO‐66 with BC(bacterial cellulose) to obtain a gel electrolyte. UIO‐66 could interact with BC through hydrogen bonds. The assembled cell with this electrolyte could operate stably at the current density of 1mAh cm^−2^, showing excellent performance. However, most reported MOF‐polymer electrolytes have the shortcomings of high impedance, which makes electrolytes unstable at high current densities. Therefore, to overcome the above shortcomings, we adopt the method of in situ composite MOF onto the electrospinning composite membrane composed of lithium alginate (LA) and polyacrylamides (PAM).^[^ ^8^ ^]^ To our knowledge, this type of MOF‐natural polymer composite electrolyte is designed and prepared for the first time through electrospinning. LA is a natural polymer, it is relatively inexpensive, easily available, and environmentally friendly compared with many reported electrostatic spinning substrate materials including polyacrylonitrile (PAN)^[^ ^2^, ^9^ ^]^ and polyvinylidene difluoride‐hexafluoro propylene copolymer (PVDF‐HFP).^[^ ^10^ ^]^ Besides, the modified LA‐based electrolyte matrix obtained by electrospinning method has a 3D network structure, which is conducive to providing the required mechanical strength of electrolyte. Moreover, ZIF‐67 can uniformly disperse on LA‐PAM electrostatic spinning membrane by the hydrogen bond interaction between imidazole groups of ZIF‐67 and the functional groups of the spinning membrane. Particularly, the rich ion transport pathways offered by ZIF‐67 and porous electrospinning membrane is not only beneficial to fast ion conduction to the uniform deposition of lithium‐ion, but also strong hydrogen bond interaction among the abundant polar functional groups in modified LA can further provide enough high mechanical strength to suppress the lithium dendrites.^[^ ^10^, ^11^ ^]^ Consequently, the assembled battery can be stably cycled at a current density of 40, 80, and even 100 mAh cm^−2^. It is worth noting that LFP cells with the MOF‐polymer composite membrane (ZIF‐67‐LA‐PAM) run steadily for 1000 cycles at 10 C, 1600 cycles at 20C for LFP cells, and 3000 h at a current density of 40 mA cm^−2^, 1300 h at 100 mA cm^−2^ for symmetric cells and the same applies to NCM 811 positive electrode and pouch cells. The superior performance of the cell implies that MOF‐natural polymer electrolyte has a very good application prospect in high‐energy‐density lithium metal batteries.

## 2 Results and Discussion

### 2.1 Preparation and Structural Characterization of Composite Membrane

**Figure 1:**
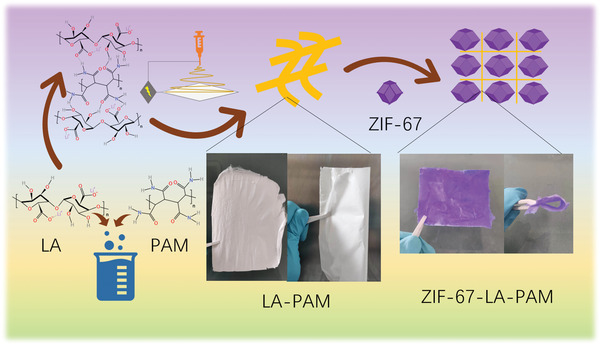
The preparation process of ZIF‐67‐LA‐PAM polymer electrolyte.

The preparation process of the MOF‐based composite solid polymer electrolyte is shown in **Figure** **1**. First, LA is prepared by the neutralization reaction of alginic acid and lithium hydroxide. Then LA and polyacrylic amide (PAM) are mixed in water in a mass ratio of 3 to 2. The LA‐PAM composite polymer is fabricated by electrospinning. Next, the ZIF‐67 is in situ synthesized on the LA‐PAM to obtain ZIF‐67‐LA‐PAM. Finally, the composite solid polymer electrolyte is prepared by adsorbing liquid electrolyte with ZIF‐67‐LA‐PAM. As shown in Figure 1 and Figure S2, Supporting Information, this type of purple membrane has good flexibility, which can be bent to accommodate flexible batteries.

**Figure 2:**
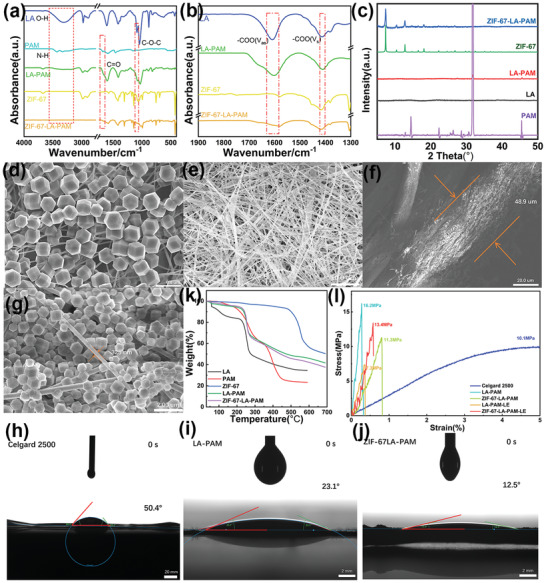
a) The FTIR spectra of LA, PAM, LA‐PAM, ZIF‐67‐LA‐PAM, and ZIF‐67. b) The FTIR spectra of LA, LA‐PAM, ZIF‐67, and ZIF‐67‐LA‐PAM in the wavenumber range from 1900–1300 cm^−1^. c) XRD patterns of electrospinning composite membranes and their components. d) The surface morphology of ZIF‐67‐LA‐PAM. e) The surface morphology of LA‐PAM. f) The cross‐section morphology of ZIF‐67‐LA‐PAM. g) The diameter of electrospinning in ZIF‐67‐LA‐PAM. h–j) The contact angles of Celgard 2500, LA‐PAM, and ZIF‐67‐LA‐PAM with liquid electrolyte at the contact time, respectively. k) TGA curves of the composite membrane and the composition. l) Stress‐strain curves and locally enlarged diagram.

To verify the existence of hydrogen bonding in ZIF‐67‐LA‐PAM, FT‐IR analysis is used to demonstrate the interaction among components including LA, PAM, and ZIF‐67. As shown in **Figure** **2**a, the characteristic absorption band of O‐H in LA appears at 3300 cm^−1^. The absorption bands of ‐NH_2_ appear at 3433 and 3340 cm^−1^. However, for LA‐PAM, only one broad weak band occurs in the range from 3300 to 3500 cm^−1^, indicating that there is a hydrogen bond interaction between the hydroxyl groups of LA and amino groups of PAM. After ZIF‐67 is in situ composited to the LA‐PAM, the characteristic absorption band above 3000 cm^−1^ can't be observed in the IR spectrum of ZIF‐67‐LA‐PAM. Besides, the C=O absorption band of PAM appears at 1676 cm^−1^ which is red‐shifted to 1658 cm^−1^ in LA‐PAM and 1643 cm^−1^ in ZIF‐67‐LA‐PAM. Moreover, the absorption band of the ether bond on the LA chain is also red‐shifted from 1089 to 1076 cm^−1^ in LA‐PAM and to 1026 cm^−1^ in ZIF‐67‐LA‐PAM. These indicate that the interaction between LA and PAM mainly comes from the hydrogen bonds between ‐NH_2_, C=O in PAM and OH, COO‐ in LA. Furthermore, it is also found from Figure 2b that the asymmetric stretching vibration band of carboxylate of LA is red‐shifted from 1608 to 1602 cm^−1^ in LA‐PAM and to 1579 cm^−1^ in ZIF‐67‐LA‐PAM, the symmetric stretching vibration band is blue‐shifted from 1411 to 1415 cm^−1^ in ZIF‐67‐LA‐PAM, revealing that there is the interaction between ZIF‐67 and LA‐PAM. Besides, theoretical calculations also confirm that the hydrogen bond is more easily formed between carboxylate radical of LA and amino group of AM (acrylamide), as well as between the nitrogen atom of the imidazole ring and the amino group of the AM, with the bond length of 2.1403 Å (Figure S33, Supporting Information). Therefore, the hydrogen bond interaction between ZIF‐67 and LA‐PAM is beneficial to forming stable composites and promoting the dissociation of LA into lithium ions. As an important property of polymer electrolytes, the crystallization behavior of polymer can influence the mechanical properties and ionic conductivity of electrolytes. X‐ray diffraction (XRD) is usually used to characterize the structure of the MOF and the crystallinity of polymer electrolytes. As shown in Figure 2c, for LA, no diffraction peak appears, indicating that LA is an amorphous state. Besides, five obvious diffraction peaks at 14.4°, 22.3°, 26.3°, 31.6°, and 45.5° are observed in PAM, which proves that PAM is easy to crystallize. However, the intensities of above‐mentioned five diffraction peaks for LA‐PAM are greatly weakened and corresponding peaks of PAM almost disappear, suggesting that the hydrogen bond interaction between LA and PAM can inhibit the crystallization of PAM. Furthermore, when ZIF‐67 is in situ synthesis onto the LA‐PAM, only characteristic diffraction peaks of ZIF‐67 occur at 7.36°, 10.4°, 12.8°, 16.5°, and 18.1°, revealing that ZIF‐67 is successfully in situ composited with LA‐PAM, as well as the crystallization of PAM is further inhibited.

Scanning electron microscopy (SEM) is applied to observe the microstructure and surface morphology of the composite solid‐state electrolyte. It can be seen from Figure 2d that massive ZIF‐67 in ZIF‐67‐LA‐PAM is observed and no agglomeration occurred between MOFs and the elemental mapping images prove that ZIF‐67 can uniformly disperse in LA‐PAM (Figure S27, Supporting Information). This may be because the hydrogen bond interaction between imidazole groups of ZIF‐67 and the hydroxyl groups or amino groups of LA‐PAM inhibits the aggregation of ZIF‐67. Besides, massive 3D pores of LA‐PAM and prismatic regular dodecahedral structure of ZIF‐67 are also observed in Figure 2d, which play an important role in absorbing a lot of liquid electrolytes. Importantly, ZIF‐67 attached to LA‐PAM can provide lithium‐ion transport pathways to facilitate lithium‐ion conduction quickly, which is beneficial to lithium evenly stripping and plating of the assembled lithium batteries. Moreover, the thickness of the ZIF‐67‐LA‐PAM is almost 48.9 µm (Figure 2f) and the diameter of electrospinning membrane LA‐PAM in ZIF‐67‐LA‐PAM is 325 nm (Figure 2g). In addition, it can be observed in Figure 2e and Figure S6, Supporting Information, the LA‐PAM is a 3D network framework with a more lager void than ZIF‐67‐LA‐PAM, while the Celgard 2500 (Figure S7, Supporting Information) is denser than that of ZIF‐67‐LA‐PAM or LA‐PAM, implying the lowest absorption capacity for the liquid electrolyte than ZIF‐67‐LA‐PAM and LA‐PAM.

### 2.2 The Adsorption Capacity for Liquid Electrolyte

To explore the influence factors of the adsorption ability of the prepared composite membrane to liquid electrolyte, contact angle test is carried out. The liquid electrolyte is dropped onto membranes and the contact angle between membranes and the liquid electrolyte is observed as shown in Figure 2h–j. The ZIF‐67‐LA‐PAM exhibits a contact angle of 12.5° with liquid electrolyte at the moment of contact and fades to 5.8° after one second (Figure S8(a), Supporting Information), which is much lower than LA‐PAM (23.1° at the moment of contact and fade to 7.6° after one second (Figure S8 (b), Supporting Information) and Celgard 2500 (50.4° at the moment of contact and fade to 39.1° after one second (Figure S8 (a), Supporting Information). It is obvious that numerous polar groups and MOFs of ZIF‐67‐LA‐PAM are beneficial to dispersing the liquid electrolyte on the composite membranes, which is attributed to the affinity between liquid electrolyte and ZIF‐67‐LA‐PAM. Therefore, the contact‐moment‐angle and after‐one‐second contact angle of LA‐PAM and ZIF‐67‐LA‐PAM are far below that of the Celgard 2500. Besides, BET test (Figure S9 and Figure S10, Supporting Information) shows that the specific surface area of ZIF‐67‐LA‐PAM increases six times after ZIF‐67 is loaded onto the electrospinning membrane LA‐PAM, and the micropores and mesoporous pores increase significantly (Figure S11, Supporting Information). Furthermore, the porosity (Table S1, Supporting Information) of ZIF‐67‐LA‐PAM can reach 88% on average, while the porosity of LA‐PAM is 53.3% on average and the porosity of the Celgard 2500 is only 37.8% on average, respectively. The rich pore structure and high porosity of ZIF‐67 promote the liquid electrolyte to soak into the ZIF‐67‐LA‐PAM and is more quickly immersed in the composite polymer electrolyte.

As described above, the liquid electrolyte is more likely to soak into composite polymer membranes with higher porosity. Higher porosity and affinity between liquid electrolytes and ZIF‐67‐LA‐PAM are key to accelerating MOF composite polymer membrane to absorb more liquid electrolytes. The absorptivity of liquid electrolytes for composite membranes and Celgard 2500 is shown in Table S2, Supporting Information. It is found that ZIF‐67‐LA‐PAM can absorb almost fivefold liquid electrolytes greatly exceeding LA‐PAM and Celgard 2500, which is good for ion fast conduction.

### 2.3 Thermal Stability and Mechanical Properties

Thermal stability and mechanical strength of the polymer electrolyte are two important factors related to its application in lithium batteries. TGA curves show the LA‐PAM and ZIF‐67‐LA‐PAM remain stable till 250 °C only 15% mass loss (Figure 2k), which can meet the requirements of thermal stability during lithium battery operation. Besides, the initial thermal decomposition temperature of the LA‐PAM is higher than that of LA and PAM, which attributes to enhancing the thermal stability by hydrogen bond interaction between LA and PAM. Unlike the ZIF‐67‐LA‐PAM, LA and PAM show significant mass loss before 200 °C. In addition, the mechanical properties of membranes are investigated by a universal testing machine. As shown in Figure 2l, the tensile strength of LA‐PAM is 16.2 MPa and its Young´s Modulus can reach 5.86 GPa (Figure S12, Supporting Information). Even though the highest tensile strength and Young´s Modulus in all membranes, the tensile strength of LA‐PAM fade to 7.3 MPa, and Young´s Modulus only reach 1.41 GPa after soaking with liquid electrolyte. On contrary, ZIF‐67‐LA‐PAM has a tensile strength of 11.3 MPa and attains a Young´s Modulus of 2.06 GPa, still getting to 13.4 MPa in tensile strength and 2.32 GPa in Young´s Modulus after a soak with liquid electrolyte. This maybe because large rigid imidazole rings of ZIF‐67 can provide mechanical strength that is still maintained even if the ZIF‐67‐LA‐PAM absorbs large amounts of liquid electrolytes. Moreover, although the strain of Celgard 2500 can reach more than 160%, its tensile strength and Young´s Modulus are lower than other membranes. It implies that the ZIF‐67‐LA‐PAM with the higher tensile strength and Young´s Modulus is more capable of inhibiting lithium dendrite growth compared to commercial separators.

### 2.4 Electrochemical Performance of Composite Membrane

**Figure 3:**
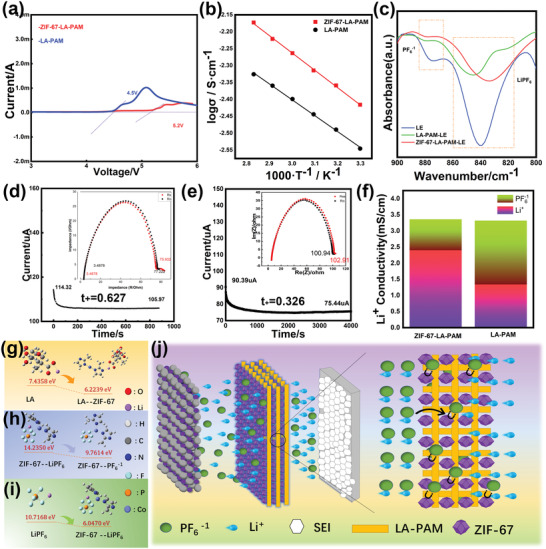
a) LSV curves of electrolytes, Lithium‐Ion Migration of ZIF‐67‐LA‐PAM. b) Temperature dependence of ionic conductivity of ZIF‐67‐LA‐PAM and LA‐PAM. c) FTIR spectra of LE, LA‐PAM, and ZIF‐67‐LA‐PAM, the lithium‐ion transfer number of the d) ZIF‐67‐LA‐PAM and e) LA‐PAM. f) The ionic conductivity of Li^+^ and PF_6_^−1^ of composite membranes at 30 °C. g) Change of binding energy of Li—O bond on LA before and after adding ZIF‐67. h) Change of Co—F bond binding energy before and after LiPF_6_ dissociation. i) Change of F—Li bond binding energy on LiPF_6_ before and after adding ZIF‐67. j) The schematic diagram of ion transport mechanism.

The electrochemical stability of the composite membranes was measured via linear sweep voltammetry (LSV) (**Figure** **3**a) and cyclic voltammetry (CV) (Figure S32, Supporting Information). Compared with electrospinning membrane LA‐PAM without ZIF‐67, the ZIF‐67‐LA‐PAM has a wider electrochemical stable window. The oxidation voltage of ZIF‐67‐LA‐PAM can reach 5.2 V, which fully meets the requirement of the LFP//Li battery and NCM811//Li battery. We further use CV to testify the electrochemical window. As shown in Figure S32, Supporting Information, there is an obvious oxidation peak of ZIF‐67‐LA‐PAM at 5.26 V, which further proves that the electrochemical stability window reaches 5.2 V and the redox peak of PF_6_^−1^ appears at 1.42 and 1.20V.^[^ ^12^ ^]^ Excellent electrochemical stability of the composite membrane is mainly due to two causes. On the one hand, the hydrogen bond interaction plays an important role in electrochemical stability. On the other hand, ZIF‐67 has a surface area of 1493.27 m^2^ g^−1^ (Figure S9, Supporting Information), which makes large amounts of impurities adsorbed to broaden the electrochemical stability window.

The ionic conductivity of electrolytes can reflect the transport capacity of ions, which can affect the performance of lithium batteries. Temperature has an important effect on the ionic conductivity of electrolyte. The dependence of ionic conductivity of composite membranes on temperature is shown in Figure 3b. It is shown that the ionic conductivities of LA‐PAM, ZIF‐67‐LA‐PAM are 2.84 and 3.84 mS cm^−1^ at 30 °C, respectively. It is also found that the ionic conductivities of the ZIF‐67‐LA‐PAM are higher than that of LA‐PAM from 30 to 80 °C. It can be attributed to the contribution of ZIF‐67 due to the absorption of more liquid electrolytes. In addition, the enhanced ionic conductivity is attributed to the improved ionic mobility of ZIF‐67‐LA‐PAM and LA‐PAM with increasing temperature. Moreover, it is found that the temperature change diagram of ionic conductivity was well fitted by the Arrhenius equation.

High lithium‐ion transference number (*t*_Li_ *^+^*) is beneficial to reducing the concentration polarization of the battery and inhibiting the formation of lithium dendrites. The *t*_Li_ *^+^* of MOF composite membrane is measured by combining AC impedance and DC polarization method. The chronoamperometry curves are shown in Figure 3d and Figure 3e and the values of *t*_Li_ *^+^* are calculated. The *t*_Li_ *^+^* of the LA‐PAM is 0.326 at 30 °C. When ZIF‐67 is composited to the LA‐PAM, the *t*_Li_ *^+^* of the ZIF‐67‐LA‐PAM rises to 0.627. It is thus obvious that the introduction of ZIF‐67 onto the LA‐PAM greatly improves the *t*_Li_ *^+^* of the electrolyte. The FTIR analysis is used to explore the effect of the ZIF‐67 in ZIF‐67‐LA‐PAM on the dissociation of lithium salt. As shown in Figure 3c, the peak of LiPF_6_ is red‐shifted from 840 to 833 cm^−1^ after ZIF‐67 is composited to LA‐PAM, suggesting that LiPF_6_ is easier to dissociate into free ions. Meanwhile, the peak of PF_6_^−1^ is blue‐shifted from 873.7 to 877.6 cm^−1^, which verifies the adsorption capacity of ZIF‐67 for PF_6_^−1^ and makes the structure more stable.^[^ ^13^ ^]^ Besides, the peak intensity of PF_6_^−1^ of ZIF‐67‐LA‐PAM‐LE is lower than that of LA‐PAM‐LE revealing that the ZIF‐67 of the 67‐LA‐PAM‐LE can absorb more PF_6_^−1^. In addition, numerous ZIF‐67 attached to LA‐PAM build a pathway to accelerate Li^+^ transport. Therefore, ZIF‐67‐LA‐PAM exhibits a higher *t*_Li_ *^+^* of 0.627(Figure 3d), while the LA‐PAM is 0.326 (Figure 3e) at 30 °C. Effective lithium ion conductivity (*σ* x *t*_Li_ *^+^*) can also reflect the lithium‐ion conduction capacity. It can be seen in Figure 3f that the effective lithium‐ion conductivity of ZIF‐67‐LA‐PAM (2.41mS cm^−1^) is higher than that of LA‐PAM (0.927mS cm^−1^) due to the higher *t*_Li+_ and higher ionic conductivity of ZIF‐67‐LA‐PAM.

Density Functional Theory (DFT) calculations are used to further confirm the effect of ZIF‐67‐LA‐PAM on the association of lithium salt. It can be seen from Figure 3g–i that the addition of ZIF‐67 onto the LA‐PAM makes the binding energy of Li—O bond in LA and F—Li bond in LiPF_6_ decrease from 7.4358 to 6.2239 eV and 10.7168 to 6.0470 eV, respectively. This demonstrates that ZIF‐67 can facilitate the dissociation of lithium carboxylate and LiPF_6_ to possess more free lithium ions in the ZIF‐67‐LA‐PAM electrolyte. Besides, it is also found in Figure 3h that the binding energy of Co—F bond decreases from 14.2350 to 9.7614 eV before and after the dissociation of LiPF_6_, which proves that ZIF‐67 can easily adsorb anions to promote the *t*_Li_ *^+^*. It can be known from the above results, lithium salts exist in three forms^[^ ^14^ ^]^ (LiPF_6_, PF_6_^−1^, and Li^+^) in the electrolyte system. Among them, PF_6_^−1^ and ZIF‐67 of ZIF‐67‐LA‐PAM are more likely to bond with each other due to lower binding energy, resulting in more dissociation of LiPF_6_ into PF_6_^−1^ and Li^+^, and the addition of ZIF‐67 leads to more dissociation of Li^+^ from LiPF_6_, resulting in higher conductivity and lithium ion transference number. As described in Figure 3j, when LiPF_6_ passes through ZIF‐67‐LA‐PAM, the LiPF_6_ is dissociated into PF_6_^−1^ and Li^+^. The PF_6_^−1^ is more easily adsorbed by Lewis acid active site of ZIF‐67 in ZIF‐67‐LA‐PAM, while Li^+^ is uniformly deposited on the surface of lithium plate through the ion transport channel provided by ZIF‐67 and 3D structural electrospinning membrane.

### 2.5 Performance of Symmetric Cells

**Figure 4:**
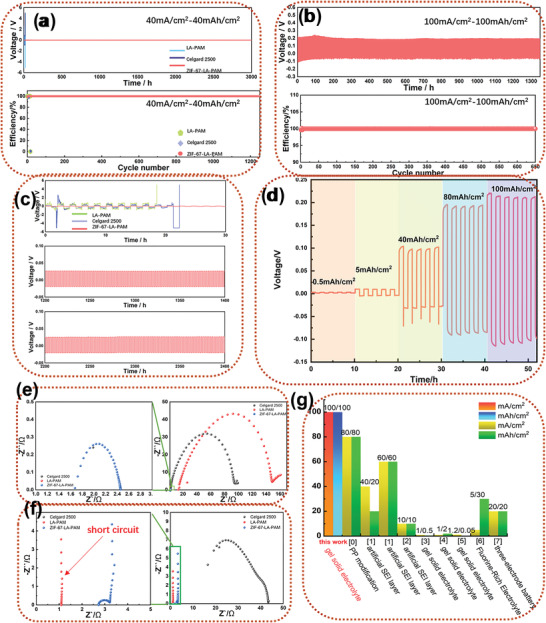
a) Symmetrical Li cells of Celgard 2500, LA‐PAM and ZIF‐67‐LA‐PAM at 40 mA cm^−2^ and c) the voltage–time curves at different times, b) symmetrical Li cells of ZIF‐67‐LA‐PAM at 100 mA cm^−2^, d) symmetrical Li cells of ZIF‐67‐LA‐PAM at different current density, the electrochemical Impedance Spectroscopy (EIS) of Celgard 2500, LA‐PAM and ZIF‐67‐LA‐PAM at 5 mA cm^−2^ after e) 0 and f) 48 h, g) Compare this work with other work including 0,^[^^]^ 1,^[^ ^15^ ^]^ 2,^[^ ^3a^ ^]^ 3,^[^ ^10a^ ^]^ 4,^[^ ^18^ ^]^ 5,^[^ ^19^ ^]^ 6,^[^ ^20^ ^]^ and 7.^[^ ^21^ ^]^

The Li//Li symmetric cells with MOFs composite membranes and Celgard 2500 were fabricated respectively to investigate the long‐term electrochemical stabilities and polarization during the Li plating and stripping process. As shown in Figure S14, Supporting Information, the symmetric cell at the current density of 5 mA cm^−2^ can run for 1600 h with a lower polarization voltage of 25 mV. Then we tried higher current density at 10 (Figure S15, Supporting Information), 20 (Figure S16, Supporting Information), 40 (**Figure** **4**a), 80 (Figure S17, Supporting Information), and even 100 mA cm^−2^ (Figure 4b). To our knowledge, the highest current density reported in the literature is only recorded to reach 60^[^ ^15^ ^]^ and 80 mA cm^−2^.^[^ ^16^ ^]^ As shown in Figure 4a, the Li/ZIF‐67‐LA‐PAM/Li cell has a stable and low polarization voltage at 26.7 mV and nearly 100% coulombic efficiency at 40 mA cm^−2^ during 3000 h. The cells run stably as well even at high current density. Concretely, even at the current density of 100 mA cm^−2^, the Li/ZIF‐67‐LA‐PAM/Li cell maintains the polarization voltage of 0.2 V for more than 1300 h. In contrast, the cells with the LA‐PAM or Celgard 2500 respectively cannot cycle stably for more than 30 h. It is worth noting that the symmetrical cell of the ZIF‐67‐LA‐PAM can run at a different current density from 0.5 to 100 mA cm^−2^(Figure 4d). Obviously, the symmetrical cell with ZIF‐67‐LA‐PAM exhibit superior cycling stability at ultrahigh current density. In order to reveal the reason, impedance tests are carried out on the symmetric battery in operation at different times. Therefore, the EIS spectra of all membranes at 5 mA cm^−2^ are shown in Figure 4e,f. Before and after batteries operation, the ZIF‐67‐LA‐PAM shows a much smaller impedance than the LA‐PAM and the Celgard 2500 (the semi‐circular arc at medium and high frequencies corresponds to the overlap of its interface impedance and carrier transfer impedance).^[^ ^17^ ^]^ After battery operation for 48 h, the ZIF‐67‐LA‐PAM appears at Warburg impedance at low frequency, which is due to the transport diffusion of lithium ions during operation. The LA‐PAM is short‐circuited during operation, resulting in a sharp decrease in impedance. Therefore, low and stable impedance guarantees low polarization voltage and stable operation at high current density. Finally, by comparison with the main results of this work with other results as shown in Figure 4g, it is obvious that ZIF‐67‐LA‐PAM has very great advantages as an electrolyte, it can operate stably at current densities up to 100 mA cm^−2^.

**Figure 5:**
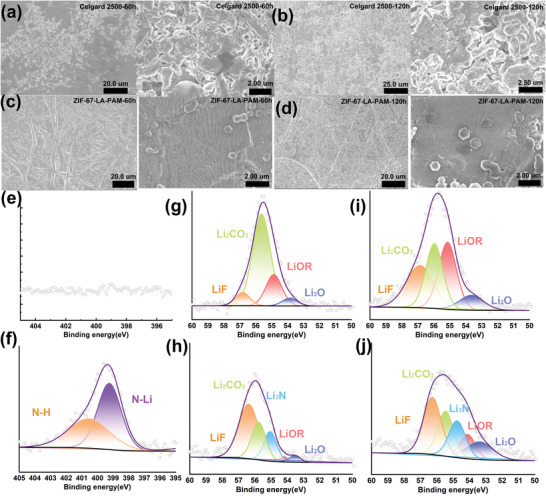
The SEM images of surface morphologies of the Celgard 2500 on the Li plat for running a) 60 and b) 120 h and the ZIF‐67‐LA‐PAM on the Li plat for running c) 60 and d) 120 h; The XPS spectra of N1s of e) Celgard 2500 and f) ZIF‐67‐LA‐PAM, Li1s of Celgard 2500 after running g) 60 and h) 120 h at 5 mA cm^−2^, Li1s of ZIF‐67‐LA‐PAM after running i) 60 and j) 120 h at 5 mA cm^−2^.

In order to further reveal the cause that the cells with ZIF‐67‐LA‐PAM have superior long‐cycling stability, the SEM is used to observe the surface morphologies of lithium metal at a current density of 5 mA cm^−2^. For Li/Celgard 2500/Li cell, the lithium dendrites grow on the surface of the lithium metal after the cell runs 60 h (**Figure** **5**a) and more lithium dendrites are observed after 120 h (Figure 5b). However, for the Li/ZIF‐67‐LA‐PAM/Li cell, no lithium dendrites but large flat sheets of lithium appear on the surface of lithium metal even if running 60 (Figure 5c) or 120 h (Figure 5d). In the Li‐Cu cell test (Figure S18, Supporting Information), the ZIF‐67‐LA‐PAM greatly reduces the nucleation overpotential of lithium(47.4 mV). It is known that the low nucleation overpotential is conducive to rapid and uniform nucleation of lithium, which verifies the inhibition ability of ZIF‐67‐LA‐PAM to the formation and growth of lithium dendrites.

The chemical composition of the solid electrolyte interphase (SEI) layer is investigated by X‐ray photoelectron spectroscopy (XPS) after running 60 h at 5 mA cm^−2^. For the Celgard 2500 battery, a large amount of Li_2_CO_3_ is detected on the surface of the lithium metal, revealing that the SEI layer is unstable which may be due to the Li_2_O reaction with air forming Li_2_CO_3_.^[^ ^22^ ^]^ On the contrary, for ZIF‐67‐LA‐PAM cells, Li_3_N appears on the surface of the lithium metal after 60 h of cycling, which can promote the stability of the SEI layer (Figure 5f). The emergence of a large amount of Li_3_N results from the reaction of amino groups of LA‐PAM and lithium metal anode, which promotes the stability of the SEI layer and rapid transport of lithium ions in the interface layer.^[^ ^23^ ^]^ In addition, ZIF‐67 effectively promotes the dissociation of LiPF_6_ and helps to promote the formation of LiF.^[^ ^24^ ^]^ The generated LiF in SEI layer can effectively promote the uniform deposition of lithium ions and inhibit the growth of lithium dendrites.^[^ ^25^ ^]^ Moreover, LiF and Li_3_N on the surface of the lithium metal increase after the cell cycling for a period of time (Figure 5h) and 120 h (Figure 5j), which suggests the formation of a more stable SEI layer.

In brief, there are three main reasons for this superior performance of the symmetric cell with ZIF‐67‐LA‐PAM electrolyte at ultrahigh current density. First of all, structural rigid of the formed ZIF‐67 layer and the hydrogen bond interaction between LA and PAM offer great robustness with very high tensile strength and Young's modulus, which can effectively prevent the growth of lithium dendrites. Second, The ZIF‐67 attached to LA‐PAM can provide a rich lithium transport pathway and soak more liquid electrolytes, which is beneficial to lithium‐ion fast conduction to improve evenly uniformed deposit the lithium‐ion. Finally, ZIF‐67‐LA‐PAM is conducive to forming a stable SEI layer between the electrolyte and metal lithium anode.

### 2.6 The Long‐Term Cycling Performance of Batteries

**Figure 6:**
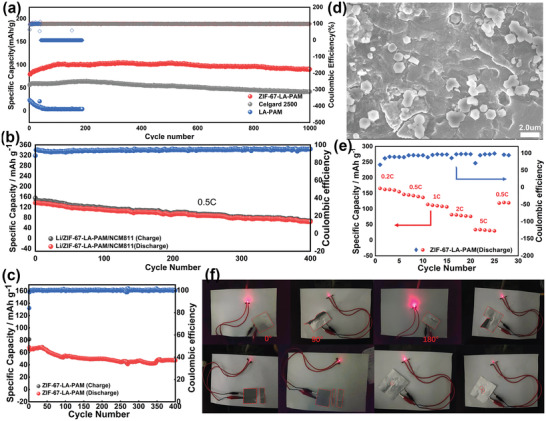
a) Long‐cycle performance of LFP cells at 10C for 1000 cycles. b) Long‐cycle performance of Li/ZIF‐67‐LA‐PAM/NCM811 cell at 0.5 C for 400 cycles. c) The pouch cell of LFP at 2C. d) The surface morphologies of lithium sheet after 800 cycles of the ZIF‐67‐LA‐PAM at 10C. e) Rate performance of Li/ZIF‐67‐LA‐PAM/LNCM811 cell. f) the LFP/ZIF‐67‐LA‐PAM/Li pouch cell powering LED bulb in parallel at the bent state, cutting, and on piercing.

We also carried out a charge and discharge cycle from 2.5 to 4 V for LFP with LiFePO_4_ as the cathode material. The LFP half‐cell shows specific capacities of 155.2, 149.6, 150, 137.7, and 120.3 mAh g^−1^ at 0.1, 0.2, 0.5, 2, and 5C, respectively (Figure S19(a), Supporting Information). Moreover, the cell with ZIF‐67‐LA‐PAM reveals excellent charge and discharge platforms with a minimal polarization voltage of 0.06 V (Figure S19(b), Supporting Information). Furthermore, a long‐time cycle with 10 C (**Figure** **6**a) is tried. It is found that the specific capacity of ZIF‐67‐LA‐PAM is higher than others. The initial discharge‐specific capacity of ZIF‐67‐LA‐PAM is 79.5mAh g^−1^ and rises to 102.7mAh g^−1^ after cycling 100 cycles. In the next 900 cycles, the cell with the ZIF‐67‐LA‐PAM always remains almost 100mAh g^−1^ and the specific capacity of the 1000 cycle is 90.8mAh g^−1^. The Coulombic efficiency is 99.2% on average and the capacity loss rate is 0.0129% per cycle. The superior long‐cycling performance of the cell is rarely reported. The rate performance of the cells at 5C is shown in Figure S20, Supporting Information. To take advantage of the wide electrochemical window of ZIF‐67‐LA‐PAM, we assemble the cells with NCM811 as the cathode, and Li metal as the anode respectively. It can be seen from Figure 6e and Figure S21, Supporting Information, that the NCM811 cell shows specific discharge capacities of 162, 142.6, 110, 78.8, and 32.3 mAh g^−1^ at 0.2, 0.5, 1, 2, and 5C, respectively. Besides, the NCM811 cell has a stable operation with an initial discharge‐specific capacity of 136.1mAh g^−1^ at 0.5C (Figure 6b). The discharge‐specific capacity is also 64mAh g^−1^ after 400 cycles with a capacity loss rate of 0.132% per cycle. It is thus clear that the cell of ZIF‐67‐LA‐PAM can be well adapted to high voltage positive electrodes and can operate stably.

Furthermore, the SEM was tested to observe the changes in Li foils after running at 10C. As shown in Figure S22(a–c), Supporting Information, the lithium dendrites growth is obviously observed for Celgard 2500 cells. In addition, dense pits (Figure S23(a,b), Supporting Information) appear on the surface of lithium sheet of the LA‐PAM cell after running for 80 cycles when the specific capacity of the cell approximately equals to zero. It can be seen that the surface of the lithium chip is eroded during the operation of the cell with the LA‐PAM, which directly leads to a sharp decrease in specific capacity. In sharp contrast, no dendrites appear for the cell with ZIF‐67‐LA‐PAM but ZIF‐67 and flat SEI layer (Figure 6d and Figure S24(a,b), Supporting Information). It may be attributed to the contribution of ZIF‐67‐LA‐PAM to promote the uniform deposition of lithium ions and the formation of stable SEI layers. Therefore, the cells with ZIF‐67‐LA‐PAM exhibit high specific capacity and long‐time operation at a high rate.

After the cell with ZIF‐67‐LA‐PAM runs at a high rate of 10C, we again explore its operating limit. It can be seen from Figure S25, Supporting Information, that the assembled LFP cell with ZIF‐67‐LA‐PAM as the electrolyte has a discharge‐specific capacity of 100mAh g^−1^ for100 cycles even at a very high rate of 20C. After running for 1600 cycles, the discharge‐specific capacity is still as high as 72.2mAh g^−1^. In contrast, the LFP battery with Celgard 2500 as the separator has decayed from 74 to 54mAh g^−1^ after 400 cycles of operation, and the discharge capacity is only 20mAh g^−1^ after 1600 cycles of operation. To our knowledge, there are few reports about the battery with long‐term stability cycling at such a high rate. It is obvious that ZIF‐67‐LA‐PAM electrolytes play a significant role in long cycling stability of the cell at ultrahigh rate.

Furthermore, we also assembled the pouch cell with ZIF‐67‐LA‐PAM and tried to test safety performance under folding, shearing, and acupuncture conditions. It is found that the pouch cell can make the LED light shine normally (Figure 6f). Meanwhile, the LFP pouch cell can run at 0.5 (Figure S26, Supporting Information) and 2C (Figure 6c). At the rate of 0.5C, the specific capacity of the pouch cell can reach 140 mAh g^−1^ for 40 cycles. Even at a high rate of 2C, the pouch cell of ZIF‐67‐LA‐PAM can reach the highest specific capacity of 69 and 47.4 mAh g^−1^ after 400 cycles with a 68.7% of capacity retention rate. As we can see from Figure S26, Supporting Information, and Figure 6c, the pouch cell with the ZIF‐67‐LA‐PAM exhibits a higher specific capacity at 0.5C, it degrades obviously after 40 cycles. However, the pouch cell can still cycle more than 400 cycles stably at 2C although it has a lower capacity, which also demonstrates the good performance of the cell with ZIF‐67‐LA‐PAM at a higher current density.

Moreover, we connected the assembled battery to the aircraft motor (Video S1, Supporting Information), and the test showed that the propeller of the aircraft can also rotate normally, exhibiting the safe and stable performance of the lithium‐ion battery with ZIF‐67‐LA‐PAM electrolyte. At last, we also tried the LFP cell of the ZIF‐67‐LA‐PAM at 0 °C (Figure S30, Supporting Information). The cell with the ZIF‐67‐LA‐PAM exhibits the highest specific capacity of 75.2mAh g^−1^ and maintains for 100 cycles at 1C, revealing that the cell with ZIF‐67‐LA‐PAM also can run at a lower temperature

All the battery tests prove that the gel polymer electrolyte we designed is not only applied to high‐rate LFP batteries, but also adapts to NCM811 cells. It is not only used for high‐rate pouch cell, but also is suitable for batteries in low‐temperature conditions.

## 3 Conclusion

In summary, a composite gel solid electrolyte ZIF‐67‐LA‐PAM was prepared by in situ synthesis of ZIF‐67 material on a modified natural polymer electrospinning film. The ZIF‐67‐LA‐PAM had excellent performance with good thermal stability and high mechanical strength due to the hydrogen interaction of LA and PAM. After in situ composite of ZIF‐67 to the LA‐PAM electrospinning membrane, the ZIF‐67‐LA‐PAM electrolyte exhibits promoted ionic conductivity, wider electrochemical stability window of 5.2 V, and higher lithium‐ion transfer of 0.627, which thanks to the contribution of ZIF‐67 of ZIF‐67‐LA‐PAM to the adsorption for anion and impurities and provide lithium‐ion conduction channels. More importantly, the ZIF‐67‐LA‐PAM electrolyte demonstrates a strong ability to inhibit lithium dendrites owing to high lithium‐ion transport capacity and formation of stable SEI layer. Consequently, the symmetric cells have superior cycling stability at ultrahigh current densities including 40, 80, and even 100 mA cm^−2^. Concretely, the Li/ZIF‐67‐LA‐PAM/Li cell has a stable and low polarization voltage at 26.7 mV and nearly 100% coulombic efficiency at 40 mA cm^−2^ for 3000 h and also maintains a stable cycling for more than 1300 h even at 100 mA cm^−2^. It is worth noting that the assembled the LFP cell, NCM811 cell, and pouch cell with the ZIF‐67‐LA‐PAM all possess superior performance. Specifically, the discharge‐specific capacity of the LFP cell can remain at 100 mAh g^−1^ for almost 900 cycles at 10C and continuous run 1600 cycles at 20C. The discharge‐specific capacity of NCM811 cell also can stably run 400 cycles. Moreover, the ZIF‐67‐LA‐PAM matches the pouch cell as well and exhibits excellent performance of safety, stability, and high performance. This study not only provides a new type of composite polymer electrolyte and a new in situ composite method of natural polymer and MOF, but also displays excellent application prospects in high‐energy‐density solid lithium batteries.

## 4 Experimental Section

### Materials

Alginic acid (Aladdin), lithium hydroxide (LiOH, Macklin), polyacrylamide (PAM, nonionic, *M_v_* = 5 000 000, Macklin), absolute methanol (Beijing TongGuang Co., Ltd), 1 m LiPF_6_ in EC& DMC& EMC (EC/DMC/EMC = 1:1:1 v/v/v, DodoChem Co., Ltd), PP Celgard 2500 separator, Cobaltous Nitrate Hexahydrate (Co(NO_3_)_2_ 6 H_2_O)(Macklin), and 2‐Methylimidazole (Macklin).

### Synthesis of Lithium Alginate (LA)

50 mmol alginic acid and 55 mmol LiOH were dissolved in 100 mL DI water to carry out a neutralization reaction. After stirring for 8 h, a large amount of methanol was added to the solution and made natural polymer LA precipitate completely. The white LA was washed twice with ethanol and then dried under vacuum at 60 °C for 12 h. The FT‐IR spectra of the carbonyl group of alginic acid and LA are shown in Figure S1, Supporting Information.

### Synthesis of LA‐PAM and ZIF‐67‐LA‐PAM

3 g LA and 2 g PAM were added into 95 mL DI water and stirred at 800 and 400r for 12 h, respectively. In the next step, the 40 mL mixture was extracted and electrospun at a high voltage of 20Kv while the distance between the needle and the collection plate was 20 cm. Air humidity needs to be less than 25%. LA‐PAM was prepared by electrospinning with an 80 mL mixture solution.

In a typical experiment, 0.2910 g (1 mmol) cobaltous nitrate hexahydrate was added into 20 mL absolute methanol and waited for the homogeneous solution. Then the LA‐PAM was soaked in the Co(NO_3_)_2_ solution and standed for 3 h. 0.3284 g (4 mmol) 2‐methylimidazole was added to 20 mL absolute methanol and put the mixture into the Co(NO_3_)_2_ solution to react for 12 h. After the reaction, the ZIF‐67‐LA‐PAM was rinsed three times with absolute methanol and vacuum dried at 60 °C for 12 h. The load of ZIF‐67 in ZIF‐67‐LA‐PAM was about 21.57 wt.%.

### Electrode Preparation and Battery Assembly

70wt% LiFePO_4_ (LFP), 20wt% Carbon Black super P, and 10wt% PVDF were ground and dispersed in N‐methyl‐2‐pyrrolidinone (NMP). 70wt% LiNi_0.8_Co_0.1_Mn_0.1_O_2_ (NCM811), 20wt% Carbon Black super P, 5wt% liquid lithium solution (LiTFSI in butanedinitrile, 0.24 mg mL^−1^), and 5wt% PVDF were ground and dispersed in N‐methyl‐2‐pyrrolidinone (NMP) too. The slurry was cast on aluminum foil and rolled alignment after drying and solidification at 60 °C for 2 h and dried at 100 °C for 12 h in a vacuum. The dried LFP and NCM811 were cut into circles with a diameter of 12 mm. Then the cathodes, composite membranes, or Celgard 2500 with liquid electrolyte and lithium metal were assembled in CR2025‐type coin cells in a glove box. All cells were working at a temperature of 30 °C.

The ZIF‐67‐LA‐PAM was soaked with liquid electrolytes before assembling the batteries. After that, the electrolyte on the surface of ZIF‐67‐LA‐PAM was drained. The electrolyte was obtained.

### Characterization

FT‐IR spectra were obtained on Bruker VERTEX 70 spectrometer with ATR mode from 400 to 4000 cm^−1^. XRD measurements were recorded on SmartLab 9Kw X‐ray diffractometer with Ni‐filtered Cu K*α* radiation in the 2*θ* range from 5° to 60°. The Hitachi SU8010 SEM produced in Japan was used to observe the structure and morphology of membranes. The surface contact angle was measured on DSA100 Kruss by the block drop method and processed by the supporting software Advance. The thermal properties of composite membranes were characterized using a TA Q600 thermogravimetric analyzer with a heating rate of 20 °C min^−1^ from 50 to 600 °C in a nitrogen atmosphere and a TA Q2000 differential scanning calorimeter with a heating rate of 10 °C min^−1^ from 90 to 150 °C. The mechanical properties of electrospinning membranes were studied using an Instron 3300 universal testing machine with an elongation rate of 3 mm min^−1^. The liquid electrolyte (1 m LiPF_6_ in EMC/DMC/EC 1:1:1, v/v/v) absorptivity of composite membranes was calculated using the following equation:

(1) $$\Delta W\left(\mathrm{wt}\%\right)=\frac{m_{s}-m_{o}}{m_{o}}\times 100\%$$

where Δ*W* is the liquid electrolyte absorptivity and *m*_o_ represents the mass of the dry membranes, *m*_s_ represents the mass of membranes after soaking the liquid electrolyte.

The porosity of membranes was figured out using the following equation:

(2) $$\Delta P\left(\%\right)=\frac{m_{1}-m_{0}}{V\times \rho }\times 100\%$$

where Δ*P* represent the Porosity of the composite electrolyte and Celgard 2500, *m*_0_ is the mass of dry membranes, and *m*_1_ represents the mass of membranes after soaking them in isopropanol. *V* is the volume of the membranes before soaking with liquid electrolyte and *ρ* is the density of isopropanol.

The MOF load was calculated by the following formula:

(3) $$\Delta L\left(\mathrm{wt}\%\right)=\frac{m_{1}-m_{0}}{m_{0}}$$

where Δ*L* is the loading of ZIF‐67 in ZIF‐67‐LA‐PAM, *m*_1_ and *m*_0_ represent the mass of ZIF‐67‐LA‐PAM and the mass of dried LA‐PAM respectively.

### Electrochemical Measurements

The electrochemical properties were characterized using a Modulab XM PhotoEchem electrochemical workstation. The electrochemical stability of electrolytes was tested by LSV by using Li/electrolyte/SS cell with a scan rate of 5 mV s^−1^ from 0 to 8 V at 30 °C. The ionic conductivity of the gel polymer electrolyte was analyzed according to the AC impedance spectra in the range of 1 Hz to 1 MHz with an AC amplitude of 5 mV by testing cells of stainless‐steel electrode (SS)/electrolyte/SS in a temperature range from 30 to 80 °C. The following equation was used to calculate the values of ionic conductivity:

(4) $$\sigma =\frac{L}{S\times R_{b}}$$

where *σ* is the ionic conductivity and L is the thickness of the electrolytes. *S* represents the contact area of SS and electrolytes while *R*_b_ is the bulk resistance of electrolytes.

To obtain the lithium‐ion transference number (*t*_Li+_) of gel polymer electrolytes, a Li/electrolyte/Li symmetrical cell was tested by chronoamperometry and AC impedance spectra at 30 °C. *t*_Li+_ is calculated according to the following equation:

(5) $$t_{\mathrm{Li}+}=\frac{I_{s}}{I_{o}}\times \frac{\Delta V-I_{o}R_{\mathrm{io}}}{\Delta V-I_{s}R_{\mathrm{is}}}$$

where Δ*V* is the polarization voltage of 10 mV and *I*_o_, *I*_s_ represents the initial state currents and the steady‐state currents respectively. *R*_io_ is the interfacial resistance before polarization and *R*_is_ is the interfacial resistance after polarization.

The compatibility of electrolytes (LFP/electrolyte/Li cell) was measured by CV with a scan rate of 0.5 mV s^−1^ from 2.5 to 4.0 V at 30 °C.

### DFT Calculations

The calculation of the bond energy between ZIF‐67 and LA and LiPF_6_ applied to DFT calculation by Gaussian 16. The PBE1PBE/6–31 g (D, P) basis group was used for all geometric optimization, and the SDD pseudopotential basis group was used for the transition metal Co. Taking the combination of ZIF‐67 and LiPF_6_ as an example, the calculation method of bond energy was as follows:

(6) $$\Delta E=E\left(\mathrm{ZIF}67\right)+E\left(\mathrm{LiPF}6\right)-E\left(\mathrm{ZIF}67-\mathrm{LiPF}6\right)$$

The higher the bond energy, the stronger the interaction.

### Molecular Dynamics

Partial atomic charges of LA and AM were derived using the restrained electrostatic potential (RESP) charge scheme, and amber atom types were matched with the GAFF^1^ database by the antechamber program of AmberTools 16 Software^2^.

The complex was dissolved in a cubic box with a side length of 24.0 Å. For each independent replica of molecular dynamics, the SHAKE algorithm was used to restrain the mobility of hydrogen atoms. The entire time interval for MD data production was 50 ns, after the performing of the minimization and equilibration stages using PMEMD.CUDA (Particle Mesh Ewald Molecular Dynamics) module integrated in AMBER 16 package.

The MD trajectories analysis for hydrogen bonds was executed through the CPPTRAJ module of Amber 16.

### CCDC

Deposition Number: 671 074^[^ ^26^ ^]^

## Conflict of Interest

The authors declare no conflict of interest.

## Supporting information

Supporting Information

Supporting Video 1

## Data Availability

The data that support the findings of this study are available from the corresponding author upon reasonable request.
