# Supplementary material for: A New In Situ Prepared MOF‐Natural Polymer Composite Electrolyte for Solid Lithium Metal Batteries with Superior High‐ Rate Capability and Long‐Term Cycling Stability at Ultrahigh Current Density
Source: Adv Sci (Weinh). 2022 Nov 15;10(3):2203916. doi: 10.1002/advs.202203916 (PMC9875675; doi:10.1002/advs.202203916)
Supplement: Supplementary file 1 — Supporting Information [file ADVS-10-2203916-s002.pdf]

Supporting Information

A New In-Situ Prepared MOF-Natural Polymer Composite Electrolyte for Solid Lithium Metal Batteries with Superior High-Rate Capability and Long-Term Cycling Stability at Ultrahigh Current Density

*Jiazhu Guan [a], Xinping Feng [a], Qinghui zeng [a], Zhenfeng Li [a], Yu Liu [a], Anqi Chen [a], Honghao Wang [a], Wei Cui [a,] *, Wei Liu [a,] *, Liaoyun Zhang [a,]**

[a] *School of Chemical Sciences, University of Chinese Academy of Sciences, Beijing 100049, China*

**Emails: zhangly@ucas.ac.cn; weiliu@ucas.ac.cn;cuiwei@ucas.ac.cn*

Table S1. The porosity of Celgard 2500, LA-PAM and ZIF-67-LA-PAM

Composite membrane	Mass before absorption (mg)	Mass after absorption (mg)	Volume (mm^3)	Porosity (%)
**Celgard 2500**	1.0	1.6	2.36	0.324
**Celgard 2500**	0.8	1.6	2.36	0.432
**LA-PAM**	0.7	3.3	5.54	0.596
**LA-PAM**	0.4	1.9	4.07	0.469
**ZIF-67-LA-PAM**	0.8	4.0	4.84	0.841
**ZIF-67-LA-PAM**	1.0	4.4	4.70	0.919

Table S2. The absorptivity of Celgard 2500 and composite polymer membranes with liquid electrolyte

Composite membrane	Mass before absorption (mg)	Mass after absorption (mg)	Absorptivity (wt%)
**PP**	4	10.5	162.5
**LA-PAM**	5.4	18.7	246.3

**ZIF-67-LA-PAM**	1.8	10.5	483.3

Table S3. the Young`s Modulus of composite membranes

Samples	Young`s Modulus (GPa)
**PP**	0.29
**LA-PAM**	5.86
**ZIF-67-LA-PAM**	1.41
**LA-PAM-LiPF$_6$**	2.06
**ZIF-67-LA-PAM-LiPF$_6$**	2.32

Table S4. The mass loading of ZIF-67

Composite membrane	LA-PAM	ZIF-67-LA-PAM	The load of ZIF-67/wt%
**Mass/ (mg)**	86.7	110.3	21.57

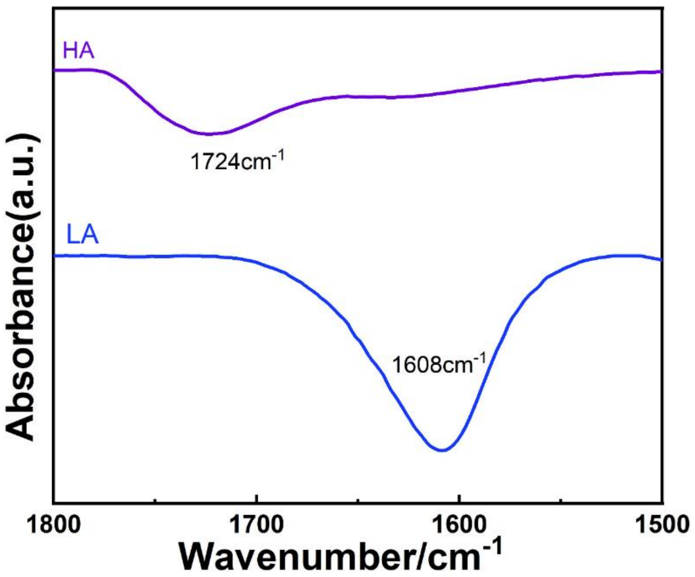

Figure S1. The FT-IR spectra of the carboxylic acid of alginate and lithium alginate.

The wavenumber of carboxylate is moving to 1608cm^{-1} from 1724cm^{-1} because of the lithiation of alginic acid[1].

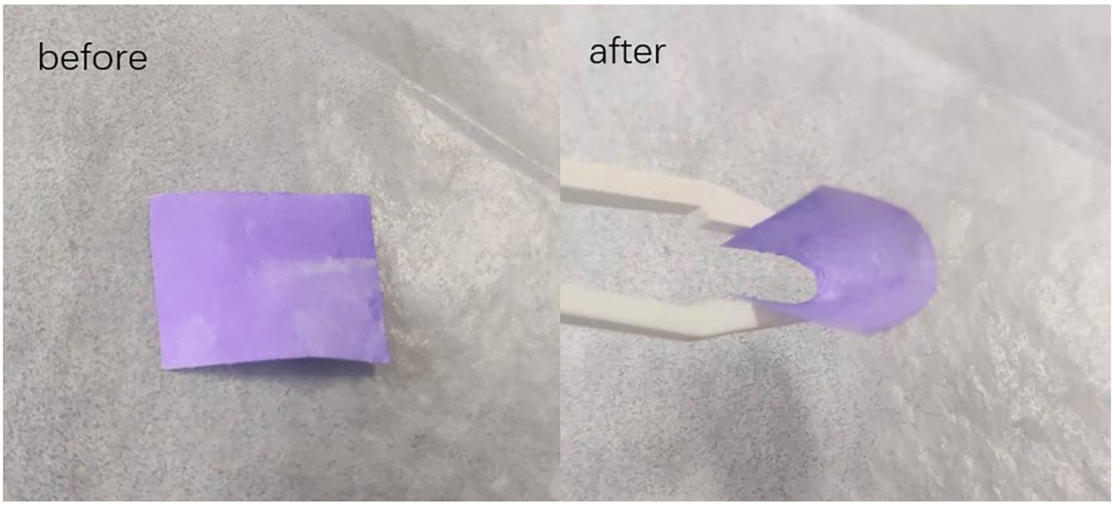

Figure S2. The flexibility test of ZIF-67-LA-PAM.

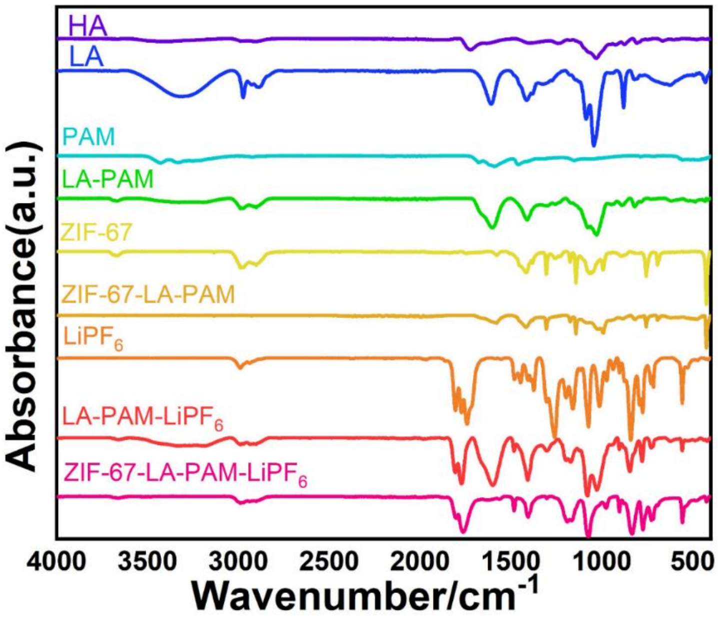

Figure S3. The FT-IR spectra of all components and composite membranes

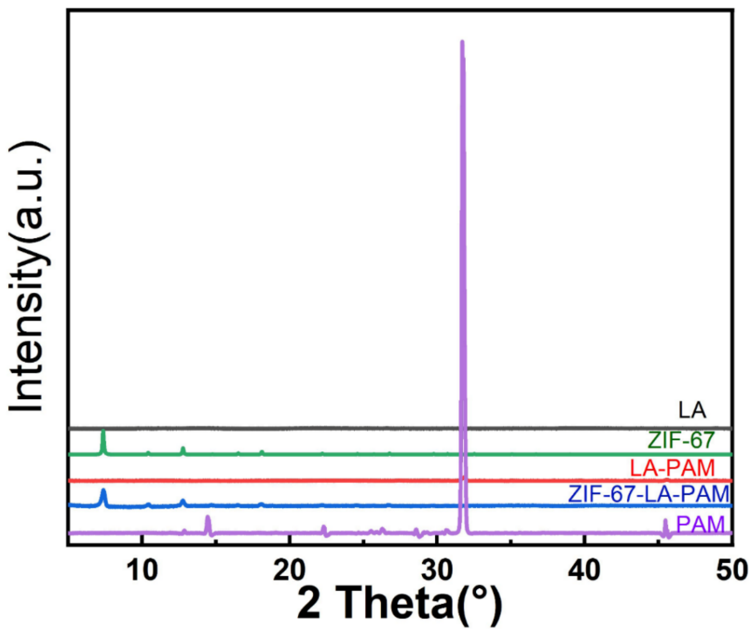

Figure S4. The XRD of all composite membranes.

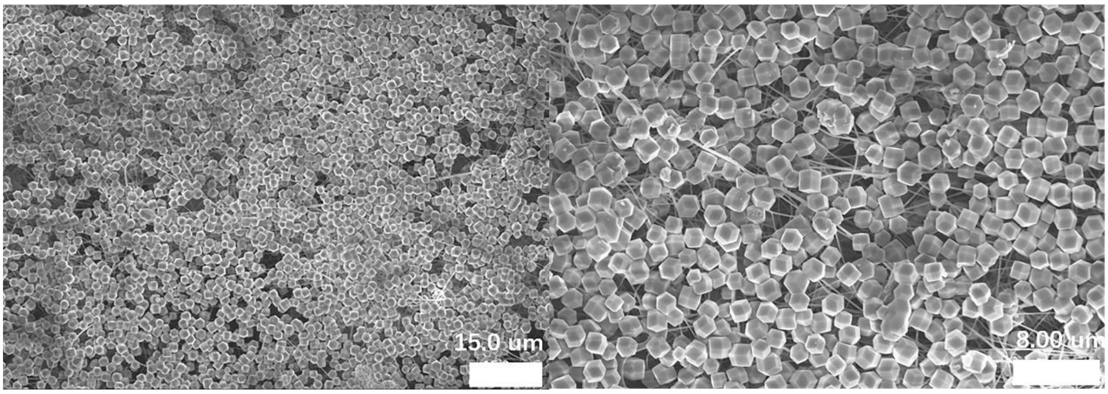

Figure S5. The surface of the ZIF-67-LA-PAM.

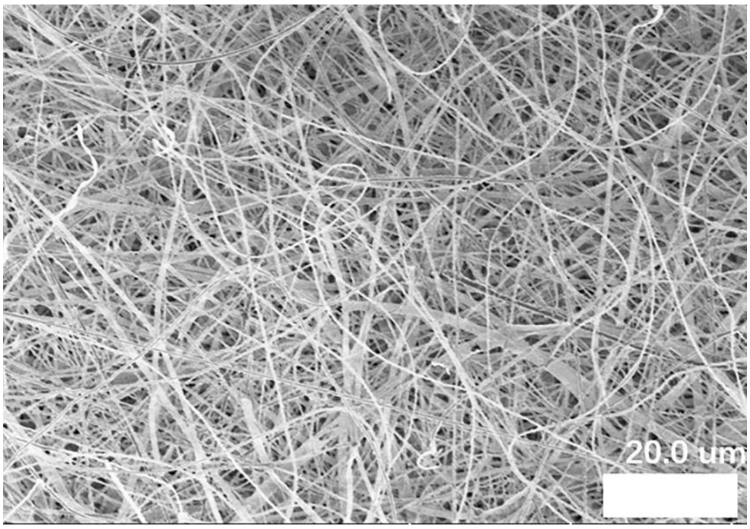

Figure S6. The surface of the LA-PAM.

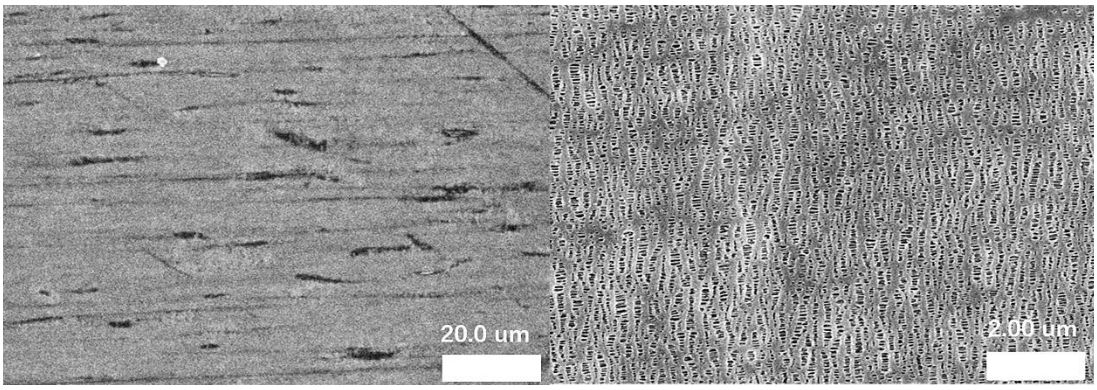

Figure S7. The surface of the Celgard 2500.

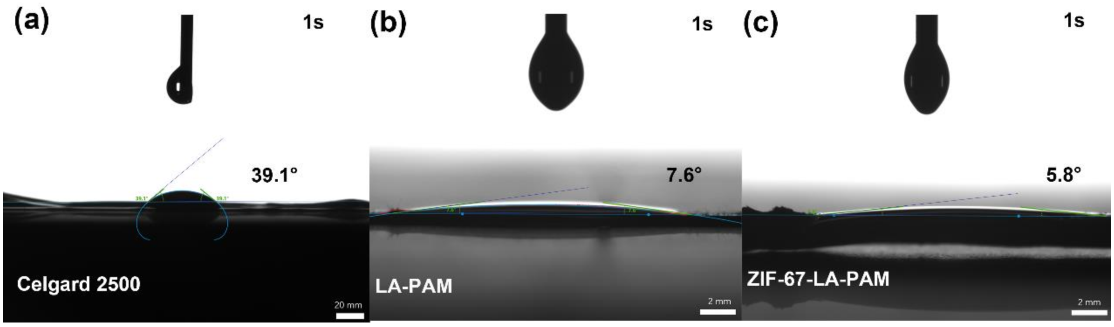

Figure S8. The contact angle of the Celgard 2500(a), LA-PAM(b) and ZIF-67-LA-PAM(c) after soaked with liquid electrolyte in 1s.

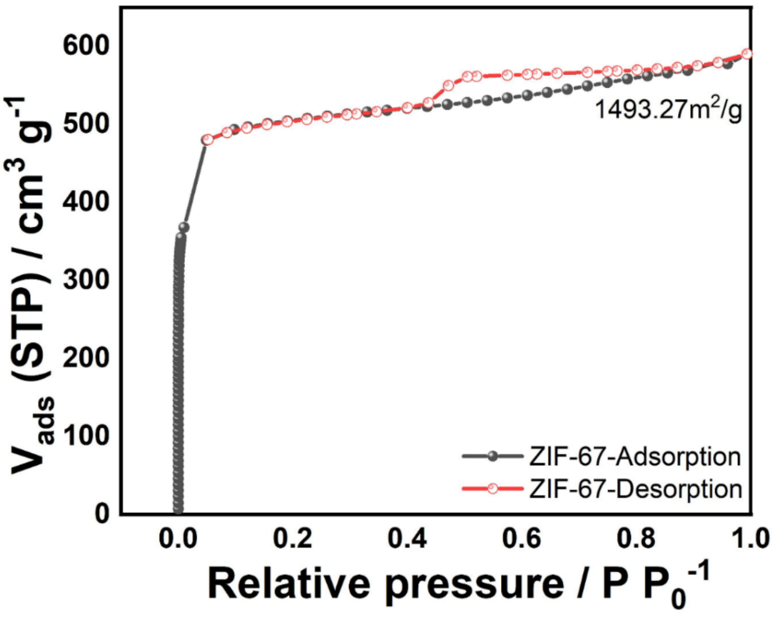

Figure S9. The specific surface area of ZIF-67.

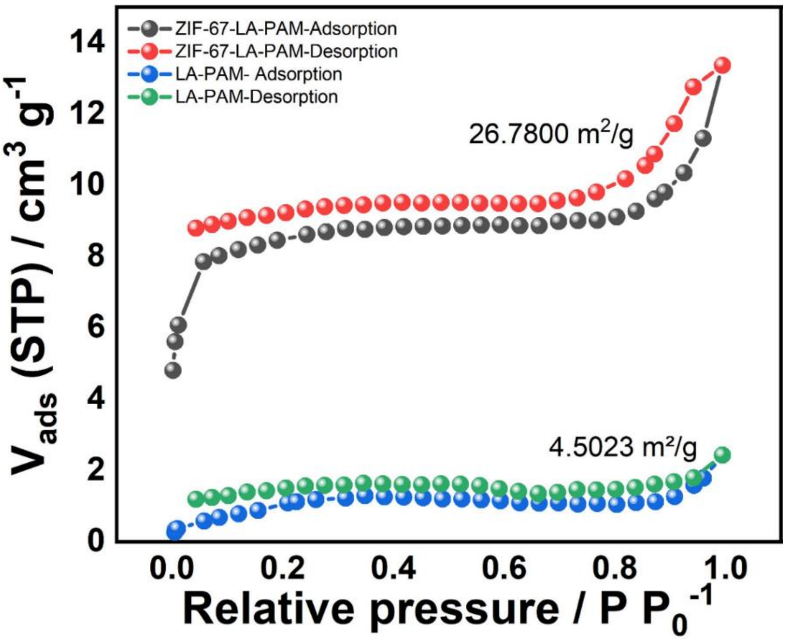

Figure S10. The specific surface areas of ZIF-67-LA-PAM and LA-PAM.

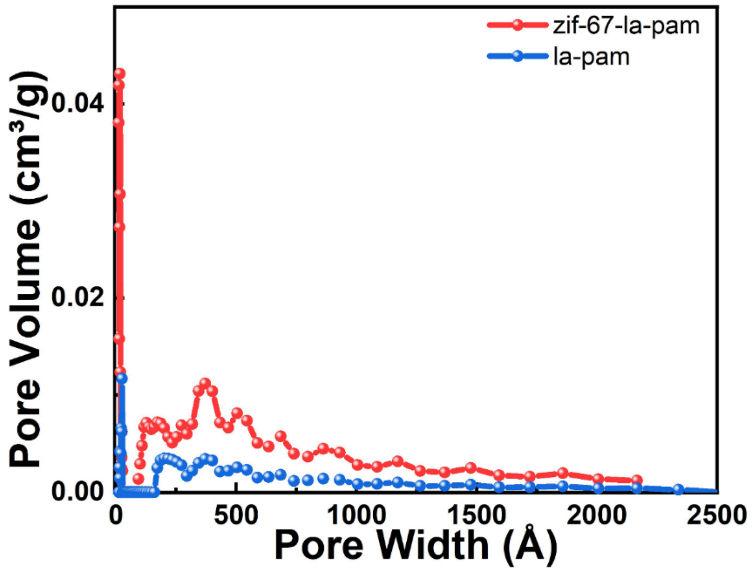

Figure S11. The pore size distribution of ZIF-67-LA-PAM and LA-PAM.

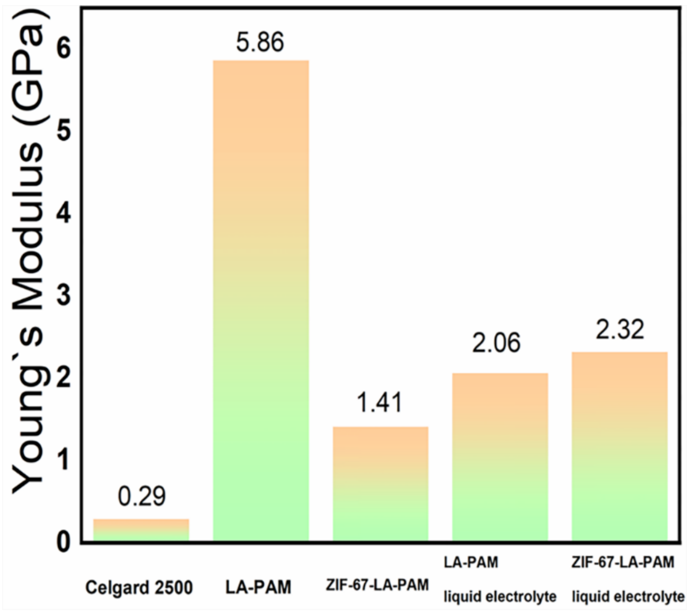

Figure S12. The tensile strength comparison diagram of electrospun composite membranes and Celgard 2500 before and after a soak with liquid electrolyte.

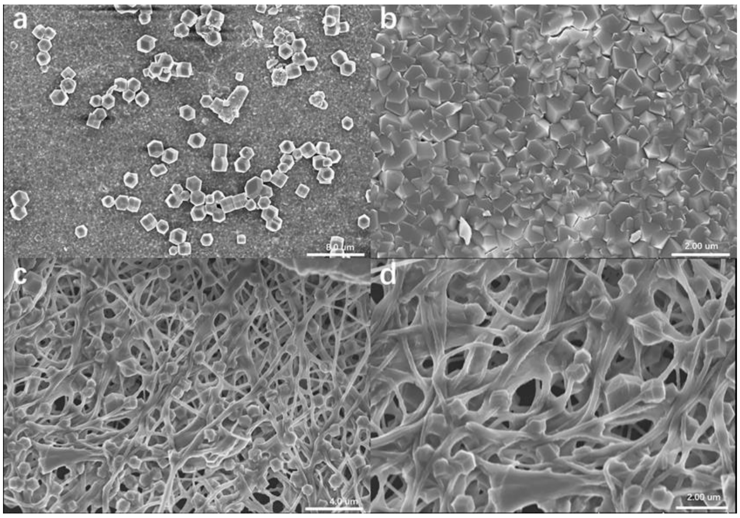

Figure S13. The morphologies of ZIF-67-LA-PAM after immersion in electrolyte

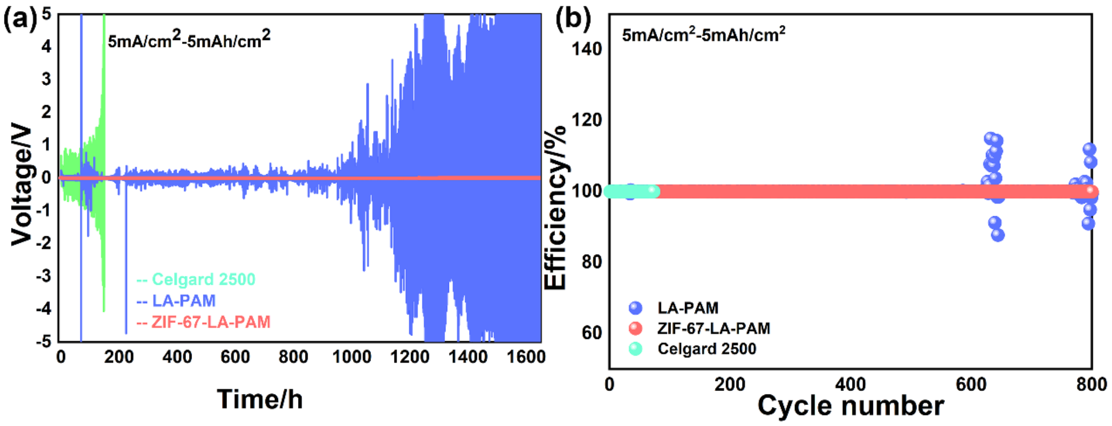

Figure S14. (a) The symmetric cell of ZIF-67-LA-PAM, Celgard 2500 and LA-PAM at 5mA/cm^2 running for 1600h; (b) The coulomb efficiency of ZIF-67-LA-PAM, Celgard 2500 and LA-PAM at 5mA/cm^2 running for 1600h.

We assembled the symmetric cell in the current density of 5 mA/cm^2 with each plating/stripping procedure of 1 h (Fig.S14a.), which showed ZIF-67-LA-PAM had a smooth operation for 1600 h at lower polarization voltage at 25 mV, while the LA-PAM and Celgard 2500 had unstable performance and higher polarization voltage. At the same time, the coulombic efficiency was 100% in the running time for ZIF-67-LA-PAM, and the coulomb efficiency of LA-PAM exceed 100% several times after 600 laps (Fig.S14 b.). The Celgard 2500 end operation because of polarization voltage out of range.

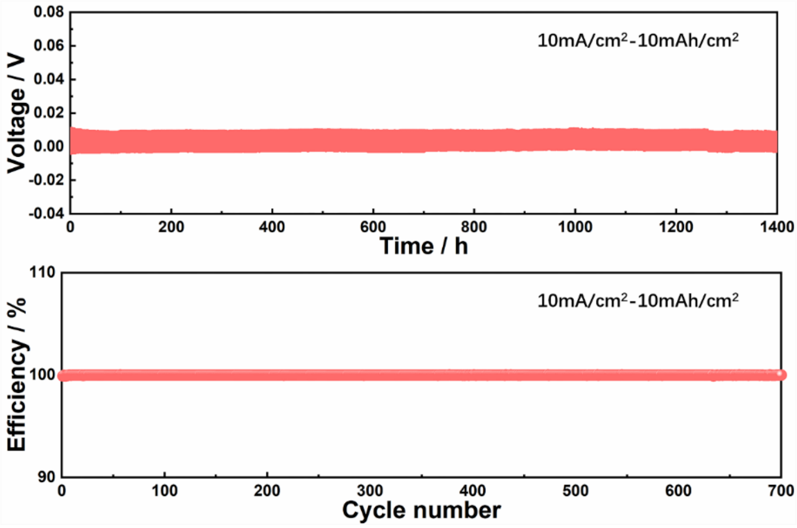

Figure S15. The symmetric cell of ZIF-67-LA-PAM at 10mA/cm^2.

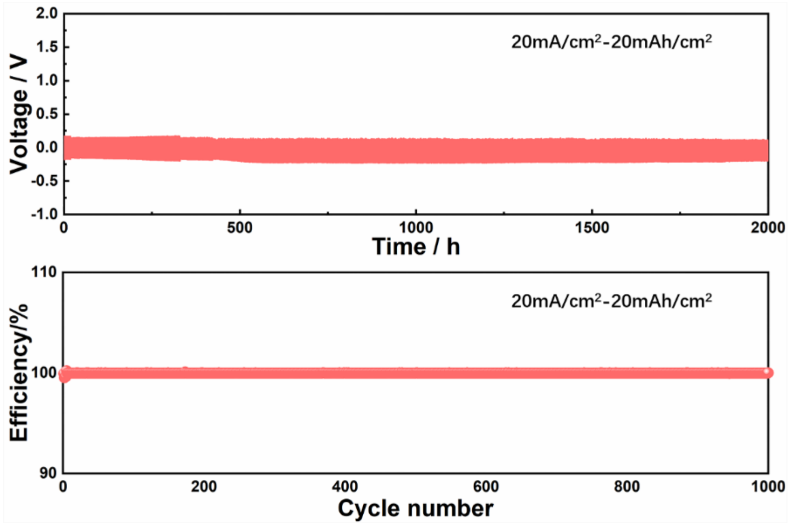

Figure S16. The symmetric cell of ZIF-67-LA-PAM at 20 mA/cm^2.

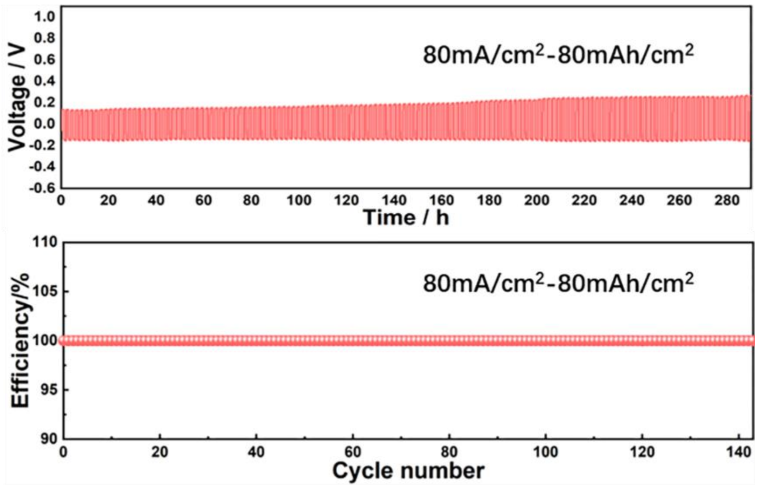

Figure S17. The symmetric cell of ZIF-67-LA-PAM at 80 mA/cm^2.

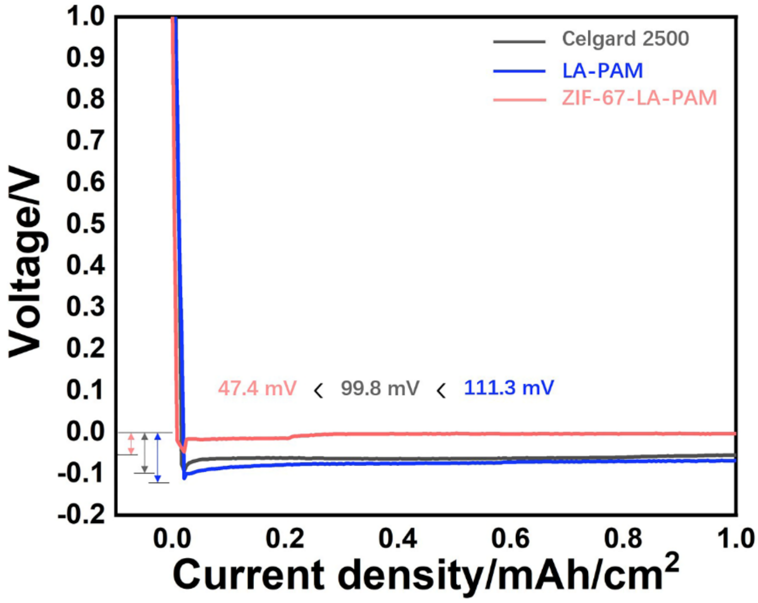

Figure S18. The nucleation overpotential of Celgard 2500、LA-PAM and ZIF-67-LA-PAM in Li-Cu cell.

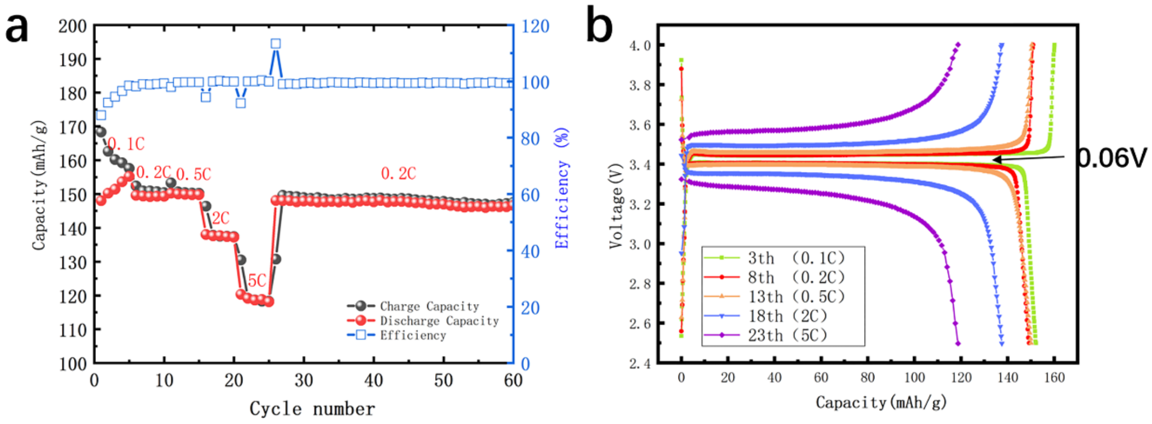

Figure S19. The LFP cells run at different rates.

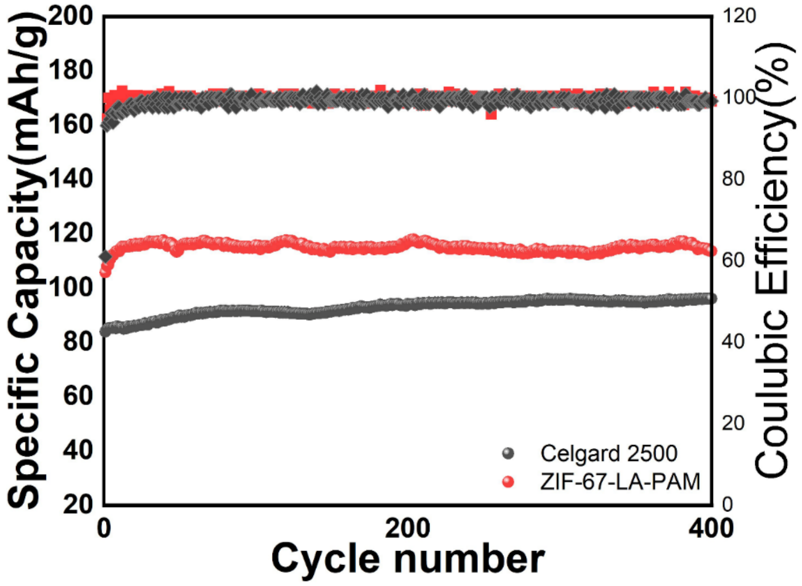

Figure S20. The LFP cells run at 5C for 400 cycles at 30℃.

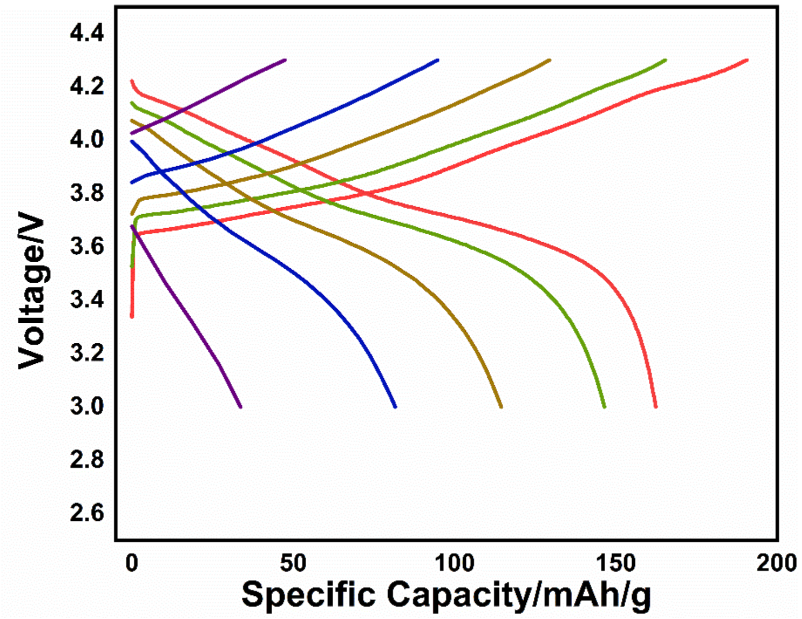

Figure S21. The NCM811 cells run at different rates at 30°C.

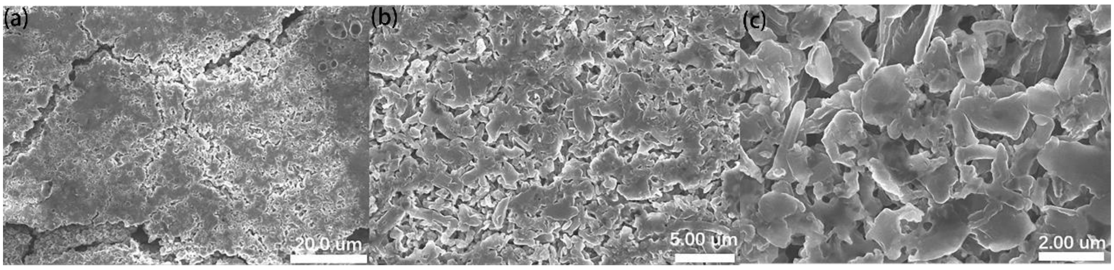

Figure S22. The surface of lithium sheet in the LFP cell with the Celgard 2500 at 10C after running 1000 cycles.

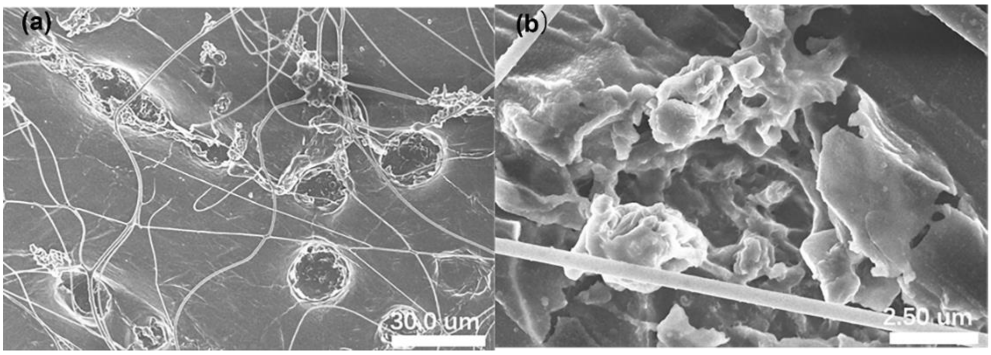

Figure S23. The surface of lithium sheet in the LFP cell with the LA-PAM at 10C after running 80 cycles.

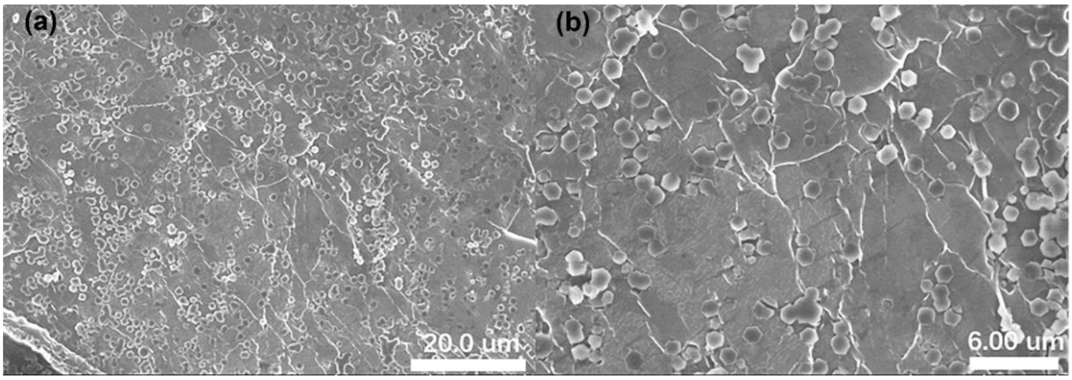

Figure S24. The surface of lithium sheet in the LFP cell with the ZIF-67-LA-PAM at 10C after running 1000 cycles.

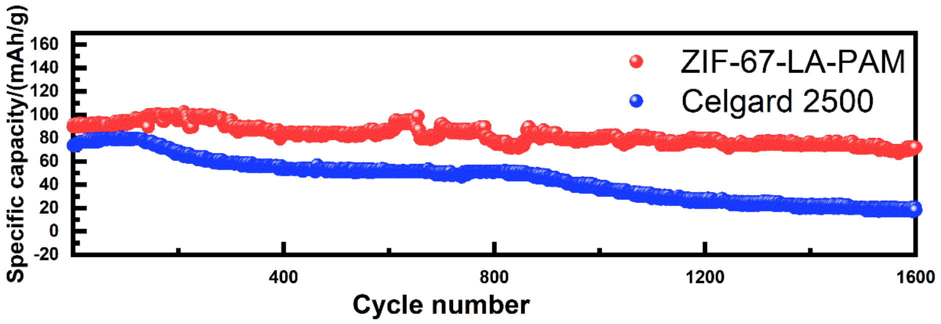

Fig S25. The LFP cells run at the rate of 20C.

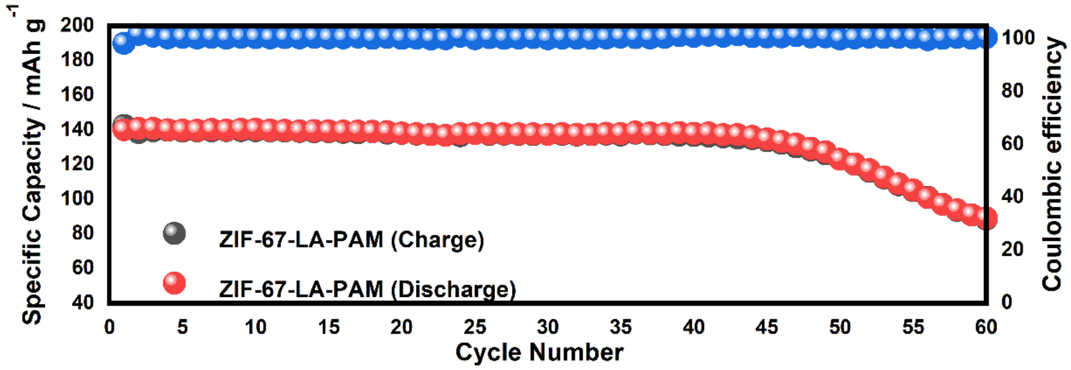

Figure S26. The LFP pouch cell runs at the rate of 0.5C with the ZIF-67-LA-PAM.

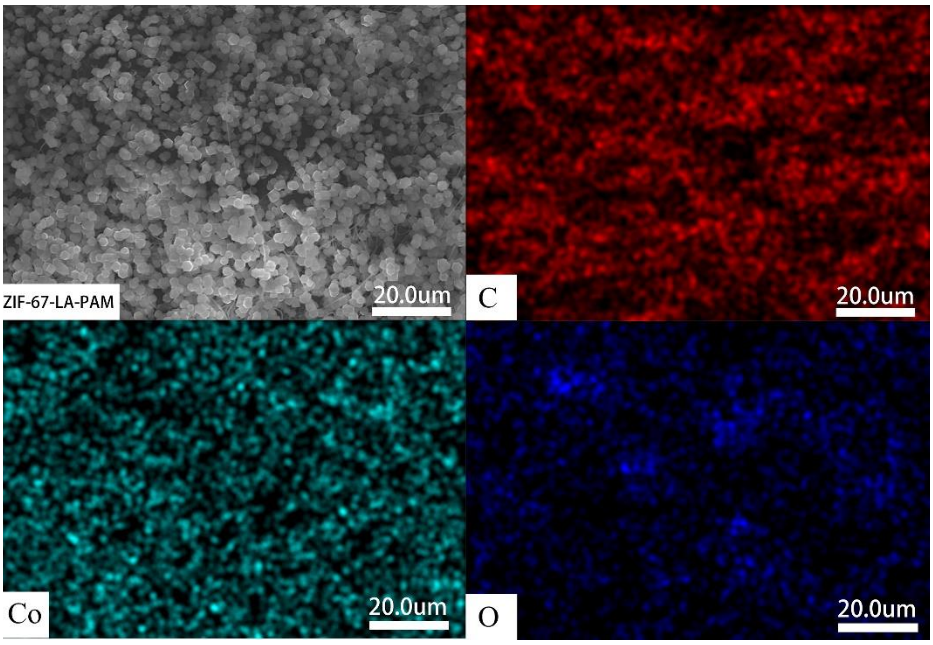

Figure S27. The element distribution on ZIF-67-LA-PAM.

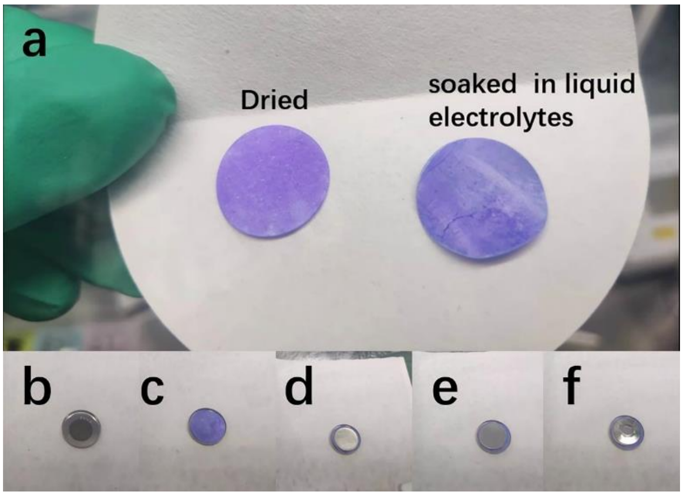

Figure S28. The picture of (a) ZIF-67-LA-PAM which dried and soaked in liquid electrolytes. (b-f) assembling cell.

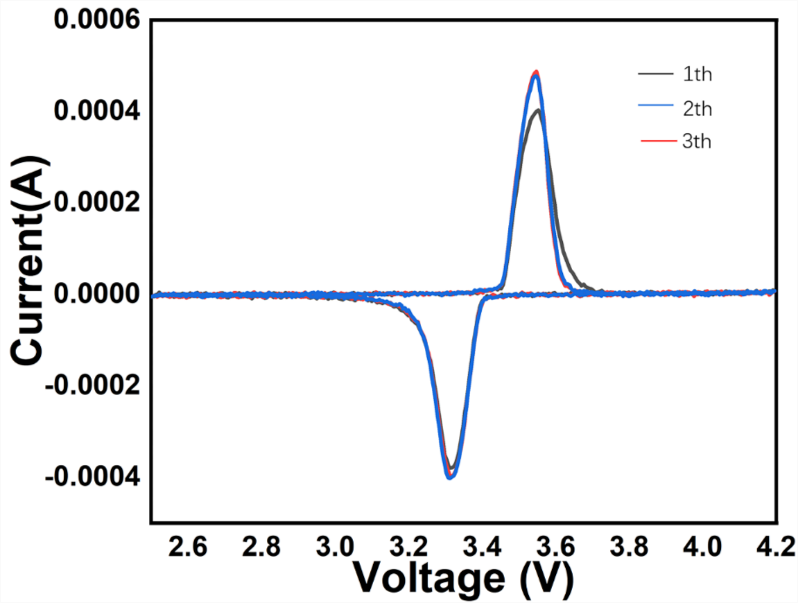

Figure S29. The cyclic voltammetry curve of ZIF-67-LA-PAM.

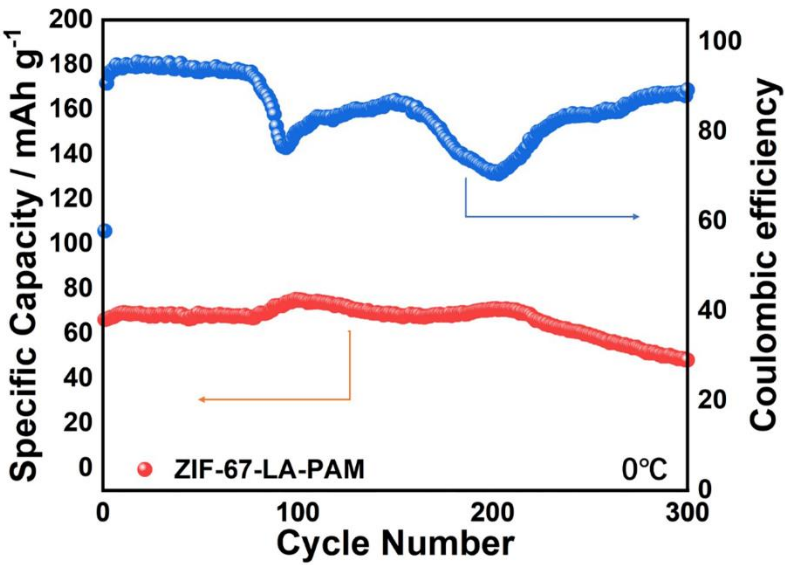

Figure S30. The LFP cell run at 1C and 0°C.

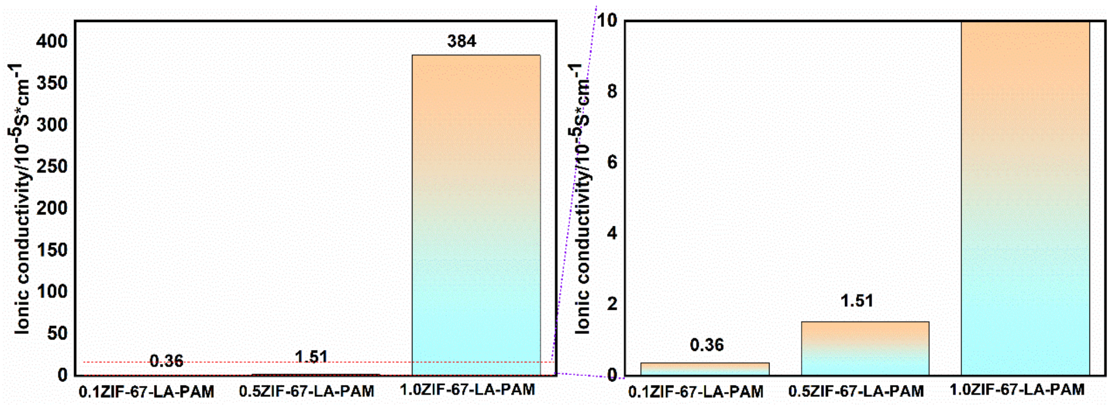

Figure S31. the EIS spectrum and ionic conductivities of different mass of the ZIF-67.

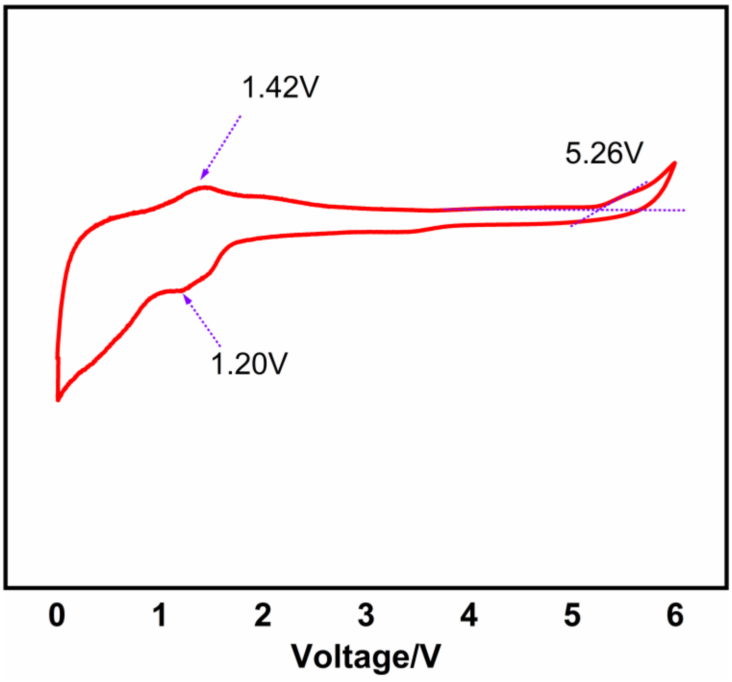

Figure S32. The CV curve of the cell with ZIF-67-LA-PAM.

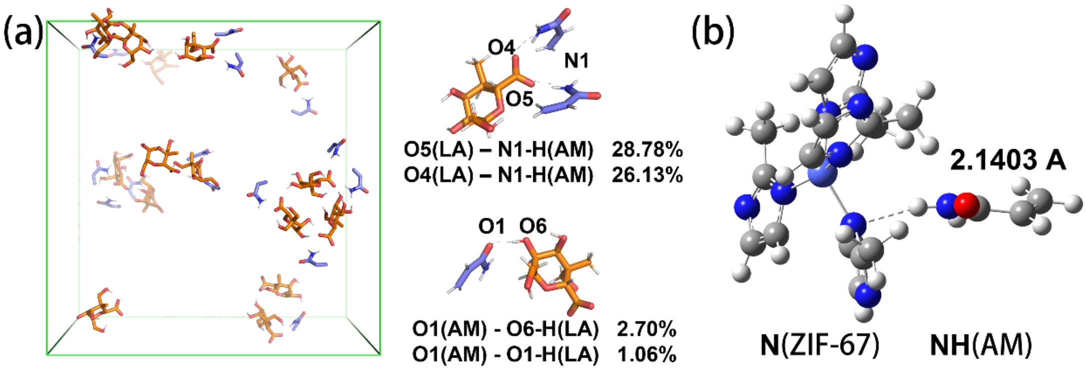

Figure S33. (a) The hydrogen interaction of LA and AM; (b) the bond length between ZIF-67 and AM.

Reference

[1] X. Wen, Q. Zeng, J. Guan, W. Wen, P. Chen, Z. Li, Y. Liu, A. Chen, X. Liu, W. Liu, S. Chen, L. Zhang, *Journal of Materials Chemistry A* **2022**, 10, 707.
